# Identification of a Novel ECM Remodeling Macrophage Subset in AKI to CKD Transition by Integrative Spatial and Single‐Cell Analysis

**DOI:** 10.1002/advs.202309752

**Published:** 2024-08-09

**Authors:** Yi‐Lin Zhang, Tao‐Tao Tang, Bin Wang, Yi Wen, Ye Feng, Qing Yin, Wei Jiang, Yue Zhang, Zuo‐Lin Li, Min Wu, Qiu‐Li Wu, Jing Song, Steven D. Crowley, Hui‐Yao Lan, Lin‐Li Lv, Bi‐Cheng Liu

**Affiliations:** ^1^ Institute of Nephrology Zhong Da Hospital Southeast University School of Medicine Nanjing Jiangsu 210009 China; ^2^ Department of Medicine Division of Nephrology Icahn School of Medicine at Mount Sinai New York NY 10029 USA; ^3^ Division of Nephrology Department of Medicine Duke University and Durham VA Medical Centers Durham NC 27705 USA; ^4^ Departments of Medicine & Therapeutics Li Ka Shing Institute of Health Sciences and Lui Che Woo Institute of Innovative Medicine The Chinese University of Hong Kong Hong Kong 999077 China

**Keywords:** AKI, CKD, macrophage, single‐cell RNA‐seq, spatial transcriptomic

## Abstract

The transition from acute kidney injury (AKI) to chronic kidney disease (CKD) is a critical clinical issue. Although previous studies have suggested macrophages as a key player in promoting inflammation and fibrosis during this transition, the heterogeneity and dynamic characterization of macrophages are still poorly understood. Here, we used integrated single‐cell RNA sequencing and spatial transcriptomic to characterize the spatiotemporal heterogeneity of macrophages in murine AKI‐to‐CKD model of unilateral ischemia‐reperfusion injury. A marked increase in macrophage infiltration at day 1 was followed by a second peak at day 14 post AKI. Spatiotemporal profiling revealed that injured tubules and macrophages co‐localized early after AKI, whereas in late chronic stages had spatial proximity to fibroblasts. Further pseudotime analysis revealed two distinct lineages of macrophages in this transition: renal resident macrophages differentiated into the pro‐repair subsets, whereas infiltrating monocyte‐derived macrophages contributed to chronic inflammation and fibrosis. A novel macrophage subset, extracellular matrix remodeling‐associated macrophages (EAMs) originating from monocytes, linked to renal fibrogenesis and communicated with fibroblasts via insulin‐like growth factors (IGF) signalling. In sum, our study identified the spatiotemporal dynamics of macrophage heterogeneity with a unique subset of EAMs in AKI‐to‐CKD transition, which could be a potential therapeutic target for preventing CKD development.

## Graphical abstract

This study sheds new light on the heterogeneous roles of macrophages in the complex and cumbersome pathological process of AKI to CKD. Integrating high‐throughput spatial and single‐cell transcriptomic data, the study identifies distinct macrophage lineages, with renal resident macrophages promoting repair and monocyte‐derived ECM remodeling macrophages (EAMs) contributing to renal fibrogenesis. These findings pave the way for the development of innovative therapeutic strategies to halt disease progression.

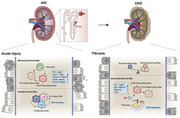

## 1 Introduction

Over 13 million people around the world are affected by acute kidney injury (AKI) each year.^[^ ^1^ ^]^ Patients with AKI are at an increased risk of developing chronic kidney disease (CKD).^[^ ^2^ ^]^ After AKI, a rapid innate immune response with inflammation is observed in the acute phase of kidney injury which involves both damage and repair processes, while persistent inflammation leads to interstitial fibrosis.^[^ ^3^, ^4^, ^5^ ^]^ Clearly, inflammation plays a key role in these processes of injury and repair during the AKI to CKD transition, but the dynamic response of immune cells and their precise contributions to this process requires clarification.

Macrophages are key regulators of immune surveillance, playing critical roles in the initiation, maintenance and resolution of tissue injury.^[^ ^6^, ^7^, ^8^ ^]^ In general, they have been classified into classical M1 and alternative M2 subtypes according to their activation states.^[^ ^9^ ^]^ Under this oversimplified, dichotomous paradigm, studies indicated that M1 macrophages generate pro‐inflammatory cytokines, exacerbating tissue damage,^[^ ^10^, ^11^ ^]^ whereas M2 macrophages promote regeneration of injured tissue, angiogenesis and matrix deposition by secreting anti‐inflammatory cytokines.^[^ ^12^ ^]^ The fine balance between M1 and M2 macrophages during immune responses affects the extent of tissue injury and repair after an acute event.^[^ ^13^ ^]^ However, this binary classification system fails to capture the dynamics of diverse plastic macrophage phenotypes and their contributions to the progression of disease. At present, different macrophage phenotypes and their activation are poorly understood in the context of AKI to CKD transition due to a lack of comprehensive knowledge regarding cell‐specific and spatiotemporal alterations in gene expression.

Recent single‐cell transcriptomic analysis has revealed unprecedented molecular details on macrophage heterogeneity.^[^ ^14^, ^15^, ^16^, ^17^ ^]^ For example, Arg1‐positive macrophages expand following early injury and promote fibrosis in human CKD.^[^ ^14^, ^16^, ^18^ ^]^ Another study identified S100A8/A9‐positive macrophages that initiate and amplify the inflammatory response during AKI.^[^ ^15^ ^]^ Therefore, macrophage heterogeneity closely correlates with kidney inflammation and fibrosis. However, the spatiotemporal dynamics of macrophage heterogeneity from AKI to CKD remain incompletely understood. Thus, there is an urgent need to identify distinct macrophage subtypes with temporal and spatial resolution to better understand this process. Here, we integrate high‐throughput spatial and single‐cell transcriptomic data that describe the spatial archetypes and macrophage heterogeneity at multiple time points during the AKI to CKD transition. We identified two major cell lineages coexisting in the ischemic kidney. Renal resident macrophages differentiated into the pro‐repair macrophage (Mac) subsets participating in wound healing, whereas monocyte derived extracellular matrix (ECM) remodeling‐associated macrophages (EAMs) transitioned toward a pro‐inflammatory Mac and contributed to chronic inflammation and kidney fibrosis. Among these clusters, EAMs communicate with fibroblasts and drive kidney fibrogenesis via Igf1‐Igf1r interactions. Thus, our data identify a new subset of macrophages with ECM remodeling capacity during the AKI to CKD transition, which could be a potential therapeutic target for preventing this disastrous disorder.

## 2 Results

### 2.1 Single‐Cell Transcriptomic Atlas of UIR Mice

**Figure 1:**
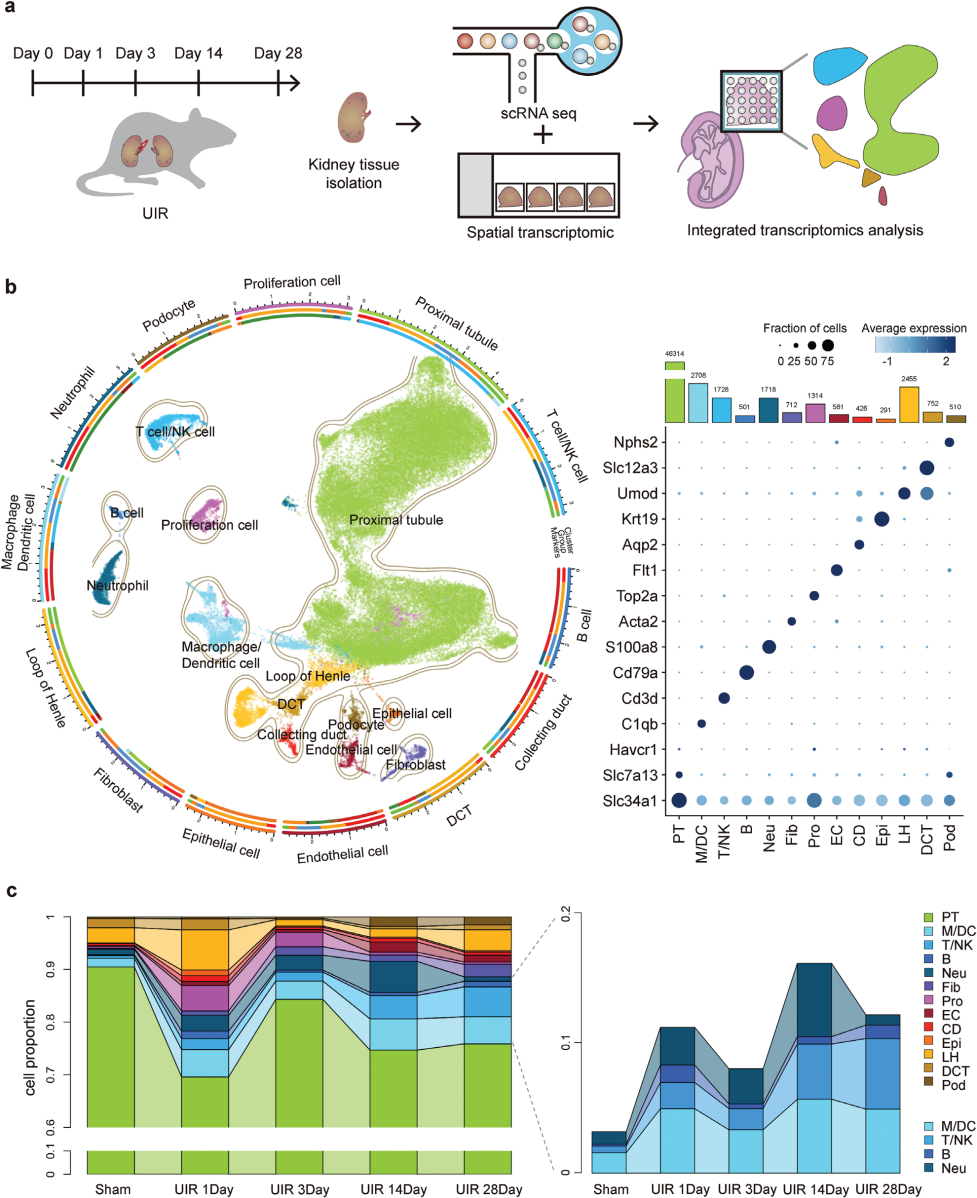
Major cell dynamics after UIR. a) The experimental workflow. Experimental AKI‐to‐CKD transition was induced in mice by UIR. Samples were collected from the injured kidneys at days 0, 1, 3, 14, and 28 post‐UIR for 10 × chromium single‐cell and visium spatial transcriptomic procedures. b) After unsupervised clustering, 2D uniform manifold approximation and projection (UMAP) visualization of the 60010 cells identified 13 major cell types. PT, proximal tubule; M/DC, monocyte, macrophage and DC; T/NK, T/NK cell; B, B cell; Neu, neutrophil; Fib, fibroblast; Pro, proliferation cell; EC, endothelial cell; Epi, epithelial cell; CD, collecting duct; DCT, Distal convoluted tubule; Pod, podocyte; LH, loop of Henle. c) Connected bar plots showing the proportionate abundance of each cell clusters in each samples. Immune cells are enlarged to facilitate data visualization.

We adopted a 35‐min warm unilateral ischemia‐reperfusion injury (UIR) technique to create the mouse AKI to CKD transition model and performed scRNA‐seq to clarify the kidney cell landscape at baseline and days 1, 3, 14, and 28 post‐UIR (**Figure** **1**a; Figure S1a, Supporting Information). We acquired a total of 74766 cells from all samples for single‐cell RNA sequencing (scRNA‐seq), with an average of 1833 genes per cell. Using the Seurat R program, cell clusters were integrated, grouped, and displayed in uniform manifold approximation and projection (UMAP) plots (Figure S1b,c, Supporting Information).^[^ ^19^ ^]^

Unsupervised clustering revealed 13 major cell clusters, encompassing proximal tubule cells (PT), fibroblasts (Fib), podocytes (Pod), and several innate immune cell types including monocyte/dendritic cells/macrophages (M/DC) (Figure 1b). Cell clusters were annotated based on the expression of curated marker genes (Figure 1b; Figure S1d, Supporting Information) and data integration with previous cell atlas resources.^[^ ^20^, ^21^, ^22^ ^]^ The most abundant cell population was PT (61.9% of total cells) which highly expressed marker genes such as *Slc34a1*, *Slc5a12*, *Slc7a13*, and *Slc22a7*. After UIR, the proportion of PT cells declined dramatically at day 1 compared to sham (69.6% vs. 90.5%) but then gradually rebounded at day 3 (84.3%), followed by a decline in the chronic phase of UIR (74.5% and 75.9% at days 14 and 28, respectively) (Figure 1c). On the contrary, the proportion of immune cells rapidly increased at day 1 (11.7%) and then reaching a peak at day 14 (16.9%) post‐UIR, which was consistent with previous reports.^[^ ^23^ ^]^ Of these, macrophages (*C1qa*, *C1qa*, and *Cd74* high) emerged as the largest, accounting 21.4% of the immune cell population, followed by T/NK cells (14.4%; *Ccl5*, *Trbc2*, and *Nkg7* high), neutrophils (13.6%; S100a8 and S100a9 high), and B cells (3.6%; Cd79a high) (Figure 1c). We also discovered a proliferative cluster with enriched expression of cell‐cycle marker genes such as *Mki67*, *Pcna*, *Top2a*, which was predominant at days 1 and 3 with a proportion of 4.8% and 2.7%, respectively (Figure 1c). This percentage decreased to 0.5% and 0.4% on days 14 and 28, respectively after injury (Figure 1c). Notably, the proliferative cluster shared marker expression with PT and Mac. Collectively, our data clearly showed the cellular atlas, especially the macrophage dynamics during AKI to CKD transition, which dominate the immune cell population.

### 2.2 Spatiotemporal Dynamics of Main Cell Types after UIR

**Figure 2:**
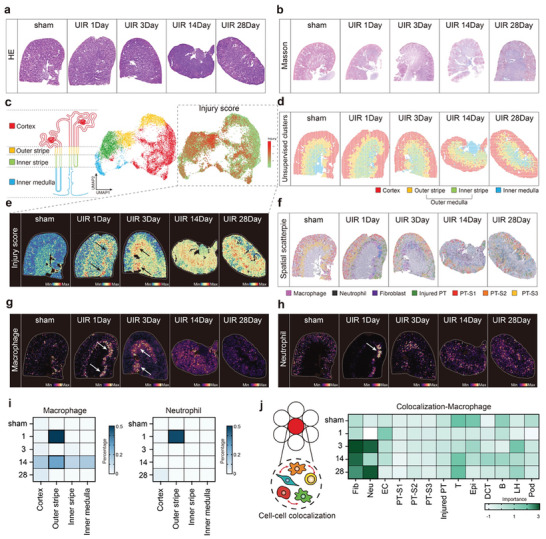
Spatiotemporal dynamics of main cell types after UIR. a) H&E staining of Visium Spatial Gene Expression samples. b) Representative Masson images at each timepoint. c,d) UMAP of spatial transcriptomics spots based on cell‐type compositions and the injury score in spatial transcriptomics. e)Injury scores in each time points. The arrows point to the areas with the highest injury score. f,g,h) The proportions of macrophages, neutrophils and multiple cells were deconvoluted from the scRNA‐seq data using the cell2location algorithm. Max, maximum; min, minimum. i) The proportion of macrophages and neutrophils infiltrated into each area according to the time‐point after UIR. j) Median relevance of cell‐type abundance in predicting other cell‐type abundances within a location.

Next, we performed spatial transcriptomic analysis on the frozen kidneys from baseline and at 1, 3, 14, and 28 days post‐UIR to uncover the spatiotemporal dynamics of renal cells during AKI progression. Masson staining revealed collagen deposition at day 3 after ischemia, which increased over time (**Figure** **2**a,b). Unsupervised clustering of transcriptomic spots from all samples based on their cell type compositions identified four clusters, which were defined as major histomorphological regions, including cortex, outer medulla (including outer and inner stripes), and inner medulla (Figure 2c,d). Injury scoring spots, calculated from the expression of inflammation and fibrosis specific genes in all samples, were present (Injury score, **Table** **1** and Methods) and further revealed the diffuse exacerbations of kidney injury during chronic stages (Figure 2e). Notably, we identified the outer stripe of the outer medulla as the site of the highest degree of injury (Figure 2c,e), consistent with the susceptibility of outer stripe to ischemic injury.^[^ ^24^ ^]^

**Table 1:** Scoring genes.

Functions	Fibrosis^[^ ^25^ ^]^	ECM^[^ ^26^, ^27^ ^]^	Inflammation^[^ ^28^, ^29^ ^]^	Injury^[^ ^25^, ^28^, ^29^ ^]^	Cytokines^[^ ^30^, ^31^, ^32^, ^33^, ^34^ ^]^	DNA repair^[^ ^35^ ^]^	Phagocytosis^[^ ^36^ ^]^	Angiogenesis^[^ ^37^ ^]^	Wound healing^[^ ^38^ ^]^
Genes	Pdgfb	Fn1	Cd74	Pdgfb	Tnf	Dctn6	Irf8	Npr3	Hps4
	Tgfb1	Spp1	Tnfrsf12a	Tgfb1	Tnfrsf1a	Bub3	Lyst	Tmem215	Arhgef19
	Col3a1	Ecm1	Cxcl1	Col3a1	Tnfrsf1b	Cenpb	Hck	Ecscr	Arhgap24
	Acta2	Mmp12	Cxcl10	Acta2	Il1b	Cenpa	Abr	Hspg2	Cldn1
	Mmp2	Mmp14	Cxcl16	Mmp2	Il6	Mki67	Lepr	Arhgap24	Fkbp10
	Pdgfa	Mmp9	Il1b	Pdgfa	Il10	Ndc80	Sirpb1a	Ang6	Gp9
	Vim	Mmp19	Cxcl2	Vim	Icam1	Cenpf	Elmo3	Ephb3	Ppara
	Nkd2	Ctsb	Ccl3	Nkd2	Vcam1	Pmf1	Met	Ephb4	Bloc1s4
	Tnc	Ctsd	Tyrobp	Tnc	Ccl5	Incenp	Mertk	Fgf9	Scnn1b
	Gpnmb	Ctsz	C3	Gpnmb	Cxcl2	Nuf2	Rab11fip2	Meis1	Scnn1g
	Cd9	Ctsl	Ccl2	Cd9	Ccl2	Smc6	Tafa4	Amotl2	Enpp4
	Tnfsf12	Ctss	Il34	Tnfsf12	Il1a	Csnk1a1	Itgb2	Ang5	Cav3
	Tl2r	Arg1	Ccl5	Tl2r	Il18	Nde1	Itgb1	Dicer1	Evpl
	Tnf	Pf4	Ccl7	Tnf	Ccl12	Cdt1	Itgam	Rnf213	Lyst
	Igf1	Thbs1	Ccl8	Igf1	Ccl7	Hells	Itgal	Sema4a	Stard13
	Timp2		Ccl12	Timp2	Ccl8	Top2a	Abca7	Sox17	Nrg1
	Anxa5		Lcn2	Anxa5	Cx3cl1	Spdl1	Lrp1	Tmem100	Hbegf
			Havcr1	Cd74	ll34	Pkhd1	Tlr4	Ereg	Ptpn6
			S100a9	Tnfrsf12a	Pdgfa	Cenpq	Anxa1	Plcd3	Serpind1
			S100a8	Cxcl1	Pdgfb	Ska3	Bltp1	Hbegf	Gp1bb
			Timp1	Cxcl10	Pdgfd	Ercc6l	Ldlr	Bcas3	Tubb1
			Cd40	Cxcl16	Tgfb2	Cbx5	Lep	Adam8	Ndnf
			Ifit1	Il1b	Ifng	Nek2	Sh3bp1	Atp5b	Myh9
			Il6	Cxcl2	Il1rn	Dync1li1	Myo7a	Ndnf	Acvrl1
			Tnf	Ccl3	Il2	Kat7	Megf10	Myh9	Proz
			Ifng	Tyrobp	Il4	Spag5	Gata2	Plxnd1	Plek
			Vcam1	C3	Il5	Kif2b	Gas6	Gab1	Chmp4b
			Icam	Ccl2	Il7	Cenps	Tusc2	Egfl7	Ajuba
			Ccl2	Il34	Il9	Sycp3	Washc5	Acvrl1	C9
			Cxcl3	Ccl5	Il12b	Nup43	Syt7	Bmpr1a	Clec10a
			Ccr2	Ccl7	Il13	Trp53bp1	Axl	Amotl1	Mertk
			Cxcr2	Ccl8	Il15	Cenpv	Mst1r	Lepr	Vps4a
			Ccn2	Ccl12	Cxcl9	Ctcf	Cebpe	Gdf2	Nog
			Trem2	Lcn2	Cxcl10	Suv39h1	Anxa3	Kdr	Pik3cb
			Cx3cr1	Havcr1	Ccl3	Ckap5	Elmo2	Jun	Kdr
			Lyz2	S100a9	Ccl4	Clasp1	Elmo1	Itgav	Itgb3
			Fcgr3	S100a8	Ltb	Rassf2	Mex3b	Itga5	Itga2b
			Ccl4	Timp1	Il16	Spc25	Elane	Itga2b	Sytl4
			Cd72	Cd40	Hmgb1	Cenpo	Cdc42se2	Rbpj	Ins2
				Ifit1	Grn	Cenpp	Slc11a1	Cemip2	Ins1
				Il6	Tnfsf10	Cenpw	Ncf2	Ovol2	Il1a
				Tnf	Cklf	Sycp1	P2ry6	Naa15	Igf1
				Ifng	Timp1	Smc3	Tyro3	Lep	Tlr4
				Vcam1	Aimp1	Zfp330	Pld4	Rora	Lox
				Icam	Cmtm3	Clasp2	Pik3ca	Wars1	Dst
				Ccl2	Tnfsf12	Knl1	Cnn2	Col24a1	Procr
				Cxcl3	Cmtm7	Septin6	Myd88	Rhoj	Ptprj
				Ccr2	Nampt	Dctn5	Anxa11	Tnfsf12	Dtnbp1
				Cxcr2	Tgfb1	Cenpl	Eif2ak1	Ramp2	Fermt3
				Ccn2	Tnfsf14	Zw10	Tub	Foxc1	Rap2b
				Trem2	Tnfsf13b	Ppp2r5c	Vav1	Gata2	Cx3cl1
				Cx3cr1	Ccl1	Cenph	Pip5k1c	Dll4	Fer1l5
				Lyz2	Il15	Hnrnpu	Dnm2	Ecm1	F10
				Fcgr3	Il16	Mis18a	Bcr	Parva	Papss2
				Ccl4	Il17a	Cenpm	Cryba1	Arhgap22	Gata4
				Cd72	Il17b	Dapk3	Tm9sf4	Fn1	Gata2
					Il17c	Birc5	Ncf4	Flt4	Gata1
					Il17d	Mis18bp1	Dock1	Flt1	Gas6
					Il17f	Ska1	Icam5	Flna	Pdpn
					Il18	Kansl1	Jmjd6	Ccn2	Fn1
					Il19	Zfp276	Rap1gap	Fgfr2	Flna
					Ccl11	Nudcd2	Tulp1	Fgfr1	Bloc1s3
					Il2	Ska2	Slamf1	Fgf6	Fgg
					Il20	Dsn1	Unc13d	Fgf2	Fgf7
					Il21	Spout1	Prtn3	Fgf1	Fgf2
					Il22	Cdca8	Fcgr2b	Htatip2	Fgf1
					Il23a	Zfp207	Nod2	Ptk2	Hgfac
					Il24	Nup85	4933434E20Rik	Epo	Erbb2
					Il25	Hsf1	Mesd	Tspan12	Epb41l4b
					Ccl12	Ss18l1	Adgrb1	Vhl	Syt11
					Il27	Cenpc1	Gulp1	Gna13	Syt7
					Ebi3	Aurkb	Rab5a	Prok1	Slc7a11
					Ifnl2	Smc5	Abl2	Pnpla6	Aqp1
					Ifnl3	Cenpx	Abl1	Ephb2	Gna13
					Il3	Ahctf1	Myo1g	Ang3	B4galt1
					Il31	Dync1i1	Cd302	Prok2	Fntb
					Il33	Anapc16	Pecam1	Adm2	Axl
					Il34	Champ1	Cdc42se1	Pten	Ccm2l
					Il4	Cenpt	Ticam2	Hand1	Hps5
					Il5	Phf6	Coro1c	Sox18	Serping1
					Il6	H3f3a	Coro1a	Nus1	Ext1
					Il7	Ppp2r5a	Pla2g5	Epas1	Ptk7
					Il9	Kat5		Epgn	Ddr1
					Lif	Cfdp1		Cspg4	Cxadr
					Lect1	Smc1a		Pank2	Grhl3
					Lect2	Lrwd1		Thsd7a	Treml1
					Lta	Stag2		Setd2	BC004004
					Csf1	Septin7		Pdcd10	P2ry12
					Mif	Nup37		Cib1	Mpig6b
					Osm	Sgo2b		Syk	Jaml
					Spp1	Rcc2		Grem1	Carmil2
					Pdgfa	Sgo1		Esm1	Anxa8
					Pdgfb	Pinx1		Ang2	Kng1
					Retn	Ngdn		Tnfaip2	Mmrn1
					Kitl	Cenpn		Casp8	Lnpk
					Thpo	Rec8		Ptk2b	Pdcd10
					Tgfa	Seh1l		Wasf2	Fgb
					Tgfb1	Dscc1		Ubp1	F11
					Tgfb2	Sgo2a		Hmox1	Arhgap35
					Tgfb3	Mad2l1		Tnfrsf12a	Myoz1
					Tnfsf11	Smc1b		Adgra2	Gpx1
					Tnfsf12	Mad1l1		Rhob	Myof
					Tnfsf13	Cenpe		Il18	Hmox1
					Tnfsf14	Septin2		Zc3h12a	Gm21974
					Ccl17	Bub1		Mmrn2	P2rx1
					Tnfsf15	Kntc1		Ptprb	Stxbp1
					Tnfsf4	Cenpk		Klf5	Stxbp3
					Tnfsf8	Bod1		Tie1	Smad4
					Tnfsf9	Tex14		Eif2ak3	Scrib
					Tnf	Plk1		Hs6st1	Slc11a1
					Xcl1	Cenpi		Prkd1	Prcp
					Fas	Sycp2l		Pofut1	Prss56
					Tnfrsf17	Rangap1		Nox1	Tspan32
					Cxcr5	Fbxo28		Apold1	Msx2
					Cd4	Dnmt3a		Gpr15	Tyro3
					Cd27	Dnmt3b		Pknox1	Mmp12
					Tnfrsf8	Fmr1		Angpt2	Vps4b
					Cd40	Dctn3		Ncl	Itgb6
					Ccr1	Itgb3bp		Kctd10	Gp6
					Ccr3	Nsmce1		Calcrl	Tfpi
					Ccr4	Spc24		Ccbe1	Gpr4
					Ccr5	Kif2c		Nr2e1	Ubash3a
					Ccr6	Uvrag		Mmp2	Nbeal2
					Ccl19	Ppp2cb		Nrp2	Celsr1
					Ccr7	Ppp2ca		Pdcl3	Krt6a
					Ccr8	Aurkc		Angpt1	Npr2
					Csf1r	Suv39h2		Amot	Notch2
					Csf2ra	Daxx		Srpx2	Nf1
					Csf2rb	Phf2		Ninj1	Plg
					Csf3r	Meikin		Ccl12	Plau
					Cx3cr1	Tpr		Med1	Serpinb2
					Epor	Ppp1cc		Dab2ip	Sdc4
					Flt3	Ndel1		Pik3ca	Sdc1
					Ccr10	Cenpu		Nppc	Cnn2
					Clcf1	Nup107		Notch1	P2ry1
					Ccl2	Knstrn		Nos3	Adipor2
					Xcr1	Zwilch		Nf1	Pard3
					Cxcr3	Zwint		Sema3e	Macf1
					Ifngr1	Cbx3		Clic4	Tfpi2
					Il1r1	Ppp2r1a		Plcd1	Serpine2
					Il2ra	Nup133		Plau	Elk3
					Il2rb	Gpatch11		Serpine1	Fzd6
					Il2rg	Rad21		Efnb2	Tgfb2
					Il3ra	Smc4		Pgf	Ap3b1
					Il4ra	Meaf6		Rtl1	F13a1
					Il5ra	Mis12		Pik3r6	Tsku
					Ccl20	Bub1b		Sirt1	Fgfr1op2
					Il6ra	Dynlt3		Elk3	Egfr
					Il6st	Stag3		Fzd8	Bnc1
					Il7r	Nsl1		Mir126b	Mia3
					Cxcr1	Hjurp		Tgfbr1	Adamts13
					Cxcr2	Cbx1		Adam15	Smpd1
					Il9r	Oip5		Plxdc1	Hpse
					Il10ra	Wdhd1		Smad5	Serpinc1
					Il12rb1	UNG		Vegfd	Apoh
					Il13ra1	SMUG1		Anpep	Cfh
					Il13ra2	MBD4		Robo4	F9
					Ccl21a	TDG		Hif3a	F8
					Il15ra	OGG1		Adgrg1	F5
					Tnfrsf9	MUTYH		Egf	F3
					Kit	NTHL1		Edn2	F2
					Lifr	MPG		Unc5b	F13b
					Ltbr	NEIL1		Mmp19	Naca
					Mst1r	NEIL2		Col15a1	Cd44
					Tnfrsf11b	NEIL3		Scg2	Cd40lg
					Tgfbr1	APEX1		Ang	Anxa5
					Tgfbr2	APEX2		Mcam	Wnt7a
					Tgfbr3	LIG3		Lemd3	Wnt5a
					Ccl22	XRCC1		Tek	Wnt3a
					Tnfrsf4	PNKP		Vav3	Vwf
					Il1r2	APLF		Cfh	Pip5k1c
					Cxcr4	HMCES		Itgb1bp1	F2rl2
					Tnfrsf25	PARP1		Angptl3	F2rl3
					Tnfrsf14	PARP2		Apln	Chmp5
					Tnfrsf18	PARP3		Xbp1	F12
					Tnfrsf11a	PARG		Wnt7b	Ccn1
					Tnfrsf10b	PARPBP		Wnt7a	Tpm1
					Osmr	MGMT		Otulin	Lrg1
					Ccr9	ALKBH2		Cyp1b1	Chmp1b
					Tnfrsf13b	ALKBH3		Pde3b	Tspan9
					Il17ra	TDP1		Vezf1	Gp1ba
					Il20ra	TDP2		Mydgf	Arl8b
					Il20rb	SPRTN		Cald1	Anxa6
					Ccr2	MSH2		Anxa2	C3
					Ccl24	MSH3		Bsg	Tor1a
					Ackr3	MSH6		Angptl6	Hif1a
					Cxcr6	MLH1		Tbx1	Ctsg
					Il11ra1	PMS2		Tal1	Crp
					Cntfr	MSH4		Bmp4	Timp1
					Il12rb2	MSH5		Epha2	Thbd
					Il23r	MLH3		Hif1a	Tgfb1
					Mpl	PMS1		Nrp1	Trim72
					Il21r	PMS2P3		Thy1	Comp
					Pdgfra	HFM1		Tgfa	Col1a1
					Pdgfrb	XPC		Col8a2	Col5a1
					Ccl25	RAD23B		Col8a1	Col3a1
					Met	CETN2		Vegfb	Ccr2
					Egfr	RAD23A		Col4a2	Hrg
					Ifngr2	XPA		Col4a1	Hnf4a
					Ifnar2	DDB1		Col18a1	Drd5
					Ifnar1	DDB2		Ccr2	Vegfa
					Il10rb	RPA1		Ccn3	Cflar
					Il22ra1	RPA2		Hrg	Yap1
					Il22ra2	RPA3		Vegfc	Entpd2
					Ifnlr1	TFIIH		Vegfa	Plec
					Ccl26	ERCC3		Meox2	Lilrb4a
					Ccl27a	ERCC2		Efna1	Eng
					Ccl3	GTF2H1		Aggf1	F2r
					Ccl4	GTF2H2		Eng	Myh10
					Ccl5	GTF2H3		Srpk2	Slc4a1
					Ccl6	GTF2H4		Nrxn1	F7
					Ccl7	GTF2H5		Nrxn3	Ano6
					Ccl8	GTF2E2		Hand2	Snai2
					Ccl9	CDK7		Fap	Dsp
					Cx3cl1	CCNH		Fmnl3	Klkb1
					Cxcl1	MNAT1		Ackr3	Entpd1
					Cxcl10	ERCC5		Rasip1	Pf4
					Cxcl11	ERCC1		S1pr1	Gp5
					Cxcl12	ERCC4		Ephb1	Cd151
					Cxcl13	LIG1		Tbx4	Pou2f3
					Cxcl14	ERCC8		Pdcd6	Shh
					Cxcl15	ERCC6		Krit1	Fga
					Cxcl16	UVSSA		Shh	Fbln1
					Cxcl17	XAB2		Shc1	Map3k1
					Cxcl2	MMS19		Shb	Dcbld2
					Cxcl3	RAD51		Ccl2	Plpp3
					Pf4	RAD51B		Apela	Cav1
					Cxcl5	RAD51D		Map3k7	Srf
					Ppbp	HELQ		Mapk14	Mrtfa
					Cxcl9	SWI5		Aimp1	Vkorc1
					Cklf	SWSAP1		Mfge8	Rab3a
					Cmtm1	ZSWIM7		Ywhaz	Hps1
					Cmtm6	SPIDR		Vav2	Dysf
					Cmtm7	PDS5B		Cav1	Proc
					Egf	DMC1		Enpep	Ppia
					Epo	XRCC2		Or10j5	Pparg
					Fgf1	XRCC3		Dysf	Alox15
					Fgf2	RAD52		Ptgs2	Adrb2
					Csf3	RAD54L		Psg22	Adrb1
					Csf2	RAD54B		Adra2b	Adra2c
					Gdf15	BRCA1		Actg1	Adra2b
					Hgf	BARD1		Ednra	Adra2a
					Ifna	ABRAXAS1		Pik3cg	Fgf10
					Ifna1	PAXIP1		Aplnr	Rab27a
					Ifna10	SHLD1		Angptl4	Chmp4c
					Ifna13	SHLD2		Prkd2	Myh2
					Ifna14	SHLD3		Optc	Plet1
					Ifna16	SEM1		Ccdc134	Bloc1s6
					Ifna2	RAD50		Cxcr3	Serpina10
					Ifna4	MRE11A		Minar1	Eppk1
					Ifna5	NBN		Epha1	Tmeff2
					Ifna6	RBBP8		Jam3	Chmp7
					Ifna7	MUS81		Becn1	Nlrp6
					Cd40lg	EME1		Ang4	Pear1
					Ifna8	EME2		Rapgef3	Pak1
					Ifnb1	SLX1A		Fgf18	C1galt1c1
					Ifne	SLX1B		Rspo3	Mustn1
					Ifng	GEN1		Ramp1	Cpb2
					Ifnk	FANCA		Vash1	Vangl2
					Il1rn	FANCB		Glul	Ubash3b
					Il1	FANCC		Angpt4	Chmp1a
					Il1a	BRCA2		Cxcl17	BC024139
					Il1b	FANCD2		Prkca	Gnas
					Cd70	FANCE		Pecam1	Chmp6
					Il1f5	FANCF		Pdgfrb	Pecam1
					Il1f10	FANCG		Pdgfa	Pdgfra
					Il10	FANCI		Col22a1	Ppl
					Il11	BRIP1		Emc10	Evl
					Il12a	FANCL		Pxdn	Pros1
					Il12b	FANCM		C1galt1	Cdkn1a
					Il13	PALB2		Fzd5	F2rl1
					Txlna	RAD51C		Prkx	Chmp2a
					Tnfsf18	SLX4			Hps6
					Mst1	FAAP20			Coro1b
					il18r1	FAAP24			Ppard
					Fasl	FAAP100			Fzd7
					Flt3l	UBE2T			Chmp2b
					Tslp	XRCC6			
					Ccl27a	XRCC5			
					Il17c	PRKDC			
					Cd40lg	LIG4			
					Tnfsf8	XRCC4			
					Crlf2	DCLRE1C			
					Sectm1a	NHEJ1			
					Bmp6	NUDT1			
					Bmp2	DUT			
					Cntf	RRM2B			
					Ccl6	PARK7			
					Ccl1	DNPH1			
					Vegfa	NUDT15			
					Il25	NUDT18			
					Ccl19	POLA1			
					Cxcl5	POLB			
					Msmp	POLD1			
					Ifna15	POLD2			
					Ccl11	POLD3			
					Il36g	POLD4			
					Pf4	POLE			
					Ppbp	POLE2			
					Pglyrp1	POLE3			
					Ifna11	POLE4			
					Gm13283	REV3L			
					Ccl25	MAD2L2			
					Cxcl3	REV1			
					Ccl21c	POLG			
					Csf1	POLH			
					Csf2	POLI			
					Ccl22	POLQ			
					Il34	POLK			
					Ccl28	POLL			
					Ccl17	POLM			
					Ccl20	POLN			
					Cxcl15	PRIMPOL			
					Cxcl13	DNTT			
					Ifne	FEN1			
					Ifna13	FAN1			
					Il36b	TREX1			
					6030468B19Rik	TREX2			
					Il1f10	EXO1			
					Ccl24	APTX			
					Ifnl3	SPO11			
					Cxcl12	ENDOV			
					Ifnab	DNA2			
					Il17b	DCLRE1A			
					Il3	DCLRE1B			
					Il12a	EXO5			
					Il27	UBE2A			
					Csf3	UBE2B			
					Tnfsf15	RAD18			
					Tnfsf13b	SHPRH			
					Ifna12	HLTf			
					Ifnl2	RNF168			
					Lta	RNF8			
					Osm	RNF4			
					Ccl9	UBE2V2			
					Il17f	UBE2N			
					Sectm1b	USP1			
					Mif	WDR48			
					Il23a	HERC2			
					Il21	H2AFX			
					Ifna14	CHAF1A			
					Il33	SETMAR			
					Ccl26	ATRX			
					Ifnk	BLM			
					Tnfsf13	RMI1			
					Ccl21a	TOP3A			
					Ccl21b	WRN			
					Xcl1	RECQL4			
					Cxcl16	ATM			
					Tnfsf4	MPLKIP			
					Il36rn	RPA4			
					Il36a	PRPF19			
					Lif	RECQL			
					Cxcl11	RECQL5			
					Il17a	RDM1			
					Ifna1	NABP2			
					Ifna2	ATR			
					Ifna4	ATRIP			
					Ifna5	MDC1			
					Ifna6	PCNA			
					Ifna7	RAD1			
					Ifna9	RAD9A			
					Ifnb1	HUS1			
					Tnfsf11	RAD17			
					Ifnz	CHEK1			
					Cxcl1	CHEK2			
					Cxcl14	TP53			
					Ifna16	RIF1			
					Cd70	TOPBP1			
					Gm13277	CLK2			
					Gm13276	PER1			
					Gm13275				
					Gm13272				
					Gm13271				
					Il17d				
					Ifna				

Since each spatial transcriptomics spot randomly captured 2–10 cells, we increased its resolution by inferring the cell‐type compositions of each spot. To accomplish this, we deconvoluted each spot using annotated scRNA‐seq data from the same sample. (Figure 2f–h; Figure S2a, Supporting Information, Table 1 and Methods). Notably, anatomical analysis showed that macrophages accumulated prominently the outer stripe area at day 1, followed by a second peak at day 14 post‐UIR, which was consistent with the scRNA‐seq data (Figure 2i). Similarly, neutrophils also accumulated in the outer stripe area at day 1 (Figure 2h and i; Figure S2a, Supporting Information). Fibroblasts emerged in limited areas and dispersed across the whole kidney with the chronic progression of fibrosis (Figure 2h; Figure S2a, Supporting Information). We next tested whether the abundances of major cell types within spots could predict the spatial dependency of macrophages with other cells during injury and repair. We evaluated colocalization, immediate and extended neighbourhood area sizes using MISTy, an explainable machine learning framework of marker interactions to profile the intra‐ and intercellular relationships (Figure 2j; Figure S2b, Supporting Information). Our findings revealed strong dependencies between macrophages, neutrophils, fibroblasts and PTs indicating that the microenvironment formed by multiple cell types may influence the dynamics of macrophage clusters. It was noted that macrophages co‐localized with neutrophils from days 3, which were remarkably co‐enriched in the outer stripe area, reflecting the recruitment of myeloid cells to injury areas early after AKI (Figure 2j). However, from day 3 onward, macrophages and fibroblasts showed stronger dependencies with each other, reflecting their close interaction in the process of fibrosis (Figure 2j).

Therefore, spatial transcriptomic data showed that macrophages and neutrophils infiltrated into the injured area at early AKI and an increased co‐localization of macrophages with fibroblasts was present from early stages to chronicity.

### 2.3 The Heterogeneity of Macrophages and Their Temporal Dynamics following UIR

**Figure 3:**
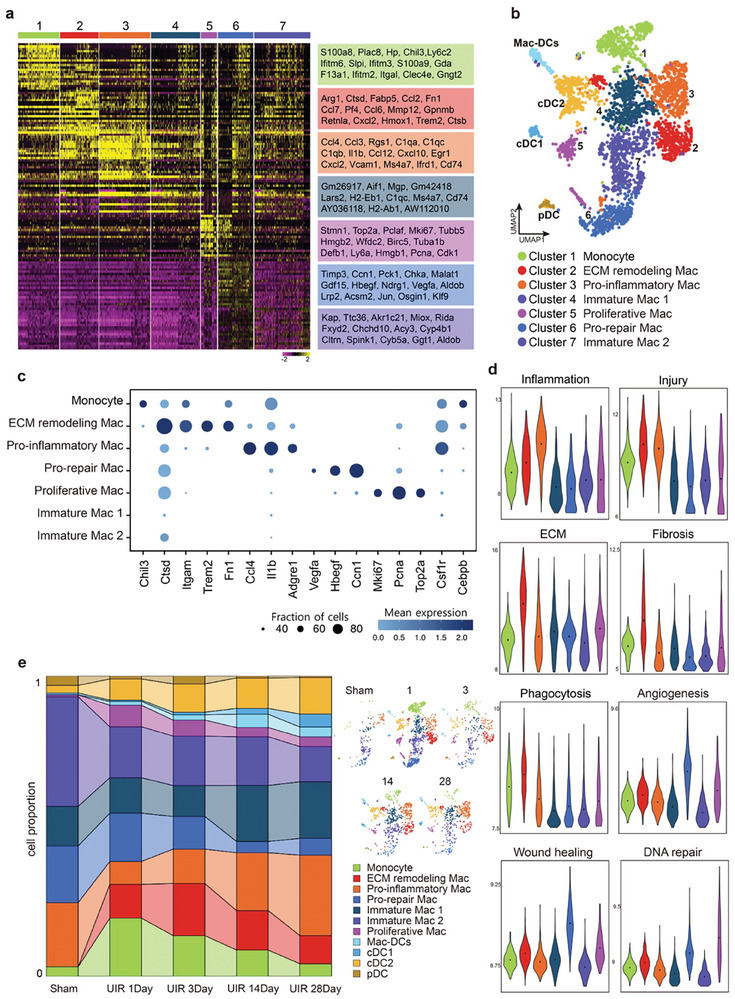
Sub‐clustering of monocyte/macrophage. a) Heatmap of top 20 marker genes in each sub‐clusters. The color scheme is based on the distribution of z‐score. b) UMAP plot of all monocyte/macrophages. c) Dot plot showing expression of marker genes. d) The score comparison of typical functions among different monocyte/macrophage clusters (all time points). e) Connected bar plots displaying the proportional abundance in each time points.

In order to gain a higher resolution of the dynamics of myeloid cell subtypes during AKI to CKD, we performed sub‐clustering analysis specifically on myeloid cells, partitioning them into 11 clusters, including monocyte (cluster 1), 6 macrophage populations (cluster 2–7) and dendritic cells (**Figure** **3**a,b). A detailed analysis revealed 7 monocyte/macrophage populations with distinct gene expression profiles (Figure 3a,c; Figure S3a, Supporting Information). Monocytes displayed the highest level of accumulation in the kidney at day 1 (Figure 3e), indicating early recruitment of monocytes to the kidney after injury. Clusters 2 and 3 expressed high levels of typical monocyte‐recruiting marker genes such as *Ccr2*, *Cx3cr1*, *Ccl2*, *Ccl3*, *Ccl4* (Figure S3a, Supporting Information) and cluster 2 was “freshly” recruited into the kidney at day 1 (Figure 3e), indicating their derivation from bone marrow‐derived monocytes.

Cluster 2 macrophages were marked by a high expression of genes encoding extracellular matrix (ECM) components (*Fn1*, *Spp1*, *Ecm1*), profibrotic genes (*Tgfb1*, *Tgfbi*, *Igf1*), metalloproteinases (*Mmp12*, *Mmp14*, *Mmp9* and *Mmp19*), and cathepsin‐encoding genes (*Ctsb*, *Ctsd*, *Ctsz*, *Ctsl* and *Ctss*) (Figure 3a,c; Figure S3a, Supporting Information), signatures associated with remodeling of the ECM. Notably, cluster 2 also expressed *Trem2*, a master regulator in sensing tissue damage, phagocytosing of apoptotic debris, and activating robust immune remodeling.^[^ ^39^ ^]^ Hence we named these cells as “ECM remodeling associated macrophages (EAMs)”.

We classified cluster 3 as “pro‐inflammatory Mac” due to their expression of inflammation‐related marker genes (*Il1b*, *Ccl3*, and *Ccl4*, *Tnf*, *Cxcl10*, *Ccl12*, and *Cxcl2*) (Figure 3a,c; Figure S3a, Supporting Information) and the highest inflammation score (Figure 3d; Figure S3c, Supporting Information). Gene ontology (GO) terms for cluster 3 was enriched in terms associated with acute inflammatory response, proinflammatory ability, and chemotaxis (Figure S3b, Supporting Information). Additionally, the proportion of this population gradually increased with AKI progression and became predominant in the late phase of UIR (days 14 and 28) (Figure 3e), indicating their potential contribution to unresolved inflammation after AKI.

Cluster 6 macrophages were present in the healthy kidney and persisted in the kidney early after UIR (Figure 3e). This population represented the homeostatic tissue‐resident macrophages according to their low expression of *Itgam* and *Fcgr1* and intermediate expression of *Adgre1*. Correspondingly, cluster 6 exhibited abundant expression of angiogenesis and wound healing genes (Figure 3a,c; Figure S3a, Supporting Information). Among the six macrophage clusters, cluster 6 had the highest wound repair and angiogenesis scores (Figure 3d; Figure S3c, Supporting Information) which was defined as “pro‐repair Mac”. Notably, the proportions of the pro‐repair cluster dropped drastically in the late chronic stages (Figure 3e), indicating depletion of pro‐repair macrophages may lead to maladaptive kidney repair after AKI.

In addition, we also identified a subset of “proliferative Mac” (cluster 5), characterized by high expression of cell‐cycle‐related genes (*Mki67*, *Top2a*, *Pcna*, *Nusap1*) (Figure 3a,c; Figure S3a, Supporting Information), which might represent the proliferative states of macrophages for replenishment after AKI. Finally, cluster 4 and 7 constituted a large proportion in homeostasis‐state and expressed low levels of macrophage terminal maturation genes such as *Csf1r*, *Cebpb*, and *Itga4* (Figure 3a,c; Figure S3a, Supporting Information), indicating a pre‐activation or immature tissue‐resident state.

Overall, the sub‐clustering analysis illustrated the dynamic heterogeneity of macrophages in the process of AKI to CKD transition. Importantly, these data revealed that pro‐inflammatory Mac contributed to the chronic inflammation, while pro‐repair Mac emerged early after AKI which continuingly declined in the chronic stage. The EAMs emerged early and persistently presented in the injured kidney, which closely correlated with ECM hemostasis throughout the entire disease process.

### 2.4 Dynamic Function of EAMs during AKI to CKD Transition

**Figure 4:**
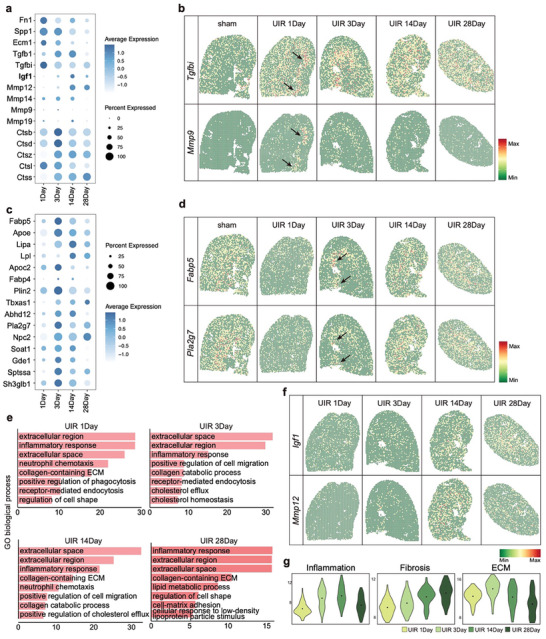
Characteristics of ECM remodeling macrophages. a) Expression of representative ECM‐related genes in EAMs after UIR. b) Gene expression of *Tgfbi* and *Mmp9* in spatial transcriptomics dataset. c) Expression of representative lipid metabolism‐related genes. d) Gene expression of *Fabp5* and *Pla2g7* in spatial transcriptomics dataset. e) Gene Ontology terms enriched from the differentially expressed genes of EAMs compared to all other Mac clusters in each time points. f) Inflammation, fibrosis and ECM scores of EAMs in each time points. g) Gene expression of *Igf1* and *Mmp12* in spatial transcriptomics dataset.

Given the unique characteristic of EAMs, we further investigated the functional significance of this cluster in the process of injury and repair after AKI. Compared to other macrophage clusters, this cluster showed dense accumulation in the outer stripe area with the highest degree of injury (Figure S2c, Supporting Information) and showed enrichment of ECM and phagocytosis related genes (**Figure** **4**a; Figure S3a, Supporting Information). One day after UIR, EAMs showed high expression of genes that promote ECM deposition as well as metalloproteinases (Figure 4a). GO terms were enriched in “extracellular region” and “collagen‐containing extracellular matrix” (Figure 4e). Notably, spatial transcriptomic analysis revealed the increasing expression of *Tgfbi* and *Mmp9* in the injured outer stripe area at day 1 post‐UIR (Figure 4b), supporting that this cluster promoted ECM deposition in the injured area, thereby facilitating tissue repair and remodeling early after AKI. By day 3 after UIR, the expression of phagocytosis and fat metabolism genes (*Fabp5*, *Trem2, Ctsb, Ctsd, Apoe*, *Lipa*, *Lpl*, *Apoc2*, *Fabp4*, *Plin2*, *Tbxas1*, *Abhd12*, *Pla2g7*, *Npc2*, *Soat1*, *Gde1*, *Sptssa*, *Sh3glb1*) were specifically upregulated in EAMs (Figure 4b,c). Visualization of these markers in our spatial transcriptomic dataset suggested enrichment of lipid‐related genes in the injured outer stripe region from day 3, highlighting its role in lipid metabolism (Figure 4d). During late chronic stages (days 14 and 28), EAMs expressed genes with well‐known pathogenic functions in fibrosis, such as *Igf1*, *Mmp12, Tgfb1*, *Tl2r*, *Tnf*, *Timp2*, *Anxa5*, *Pdgfa*, and exhibited the highest fibrosis score at day 14 (Figure 4b). High levels of *Mmp12*, *Mmp9* were observed in this population, which could diminish matrix degradation and augment fibrotic responses. Additionally, remarkable expression of *Igf1*, encoding a member of the insulin‐like growth factor (IGF) family of proteins known to promote myofibroblast survival,^[^ ^40^ ^]^ was gradually increased and specifically expressed in macrophages (Figure 4g). Collective scoring analysis showed that this population was characterized with a high ECM score at early stages after ischemia (days 1 and 3), as well as a high fibrosis score at chronic stages (days 14 and 28) (Figure 4f).

We thus conclude that EAMs appeared early after AKI and persist into the chronic stages, which participated in the dynamic process of ECM remodeling during AKI to CKD transition.

### 2.5 Lineage Analysis of Mononuclear Phagocytic System

**Figure 5:**
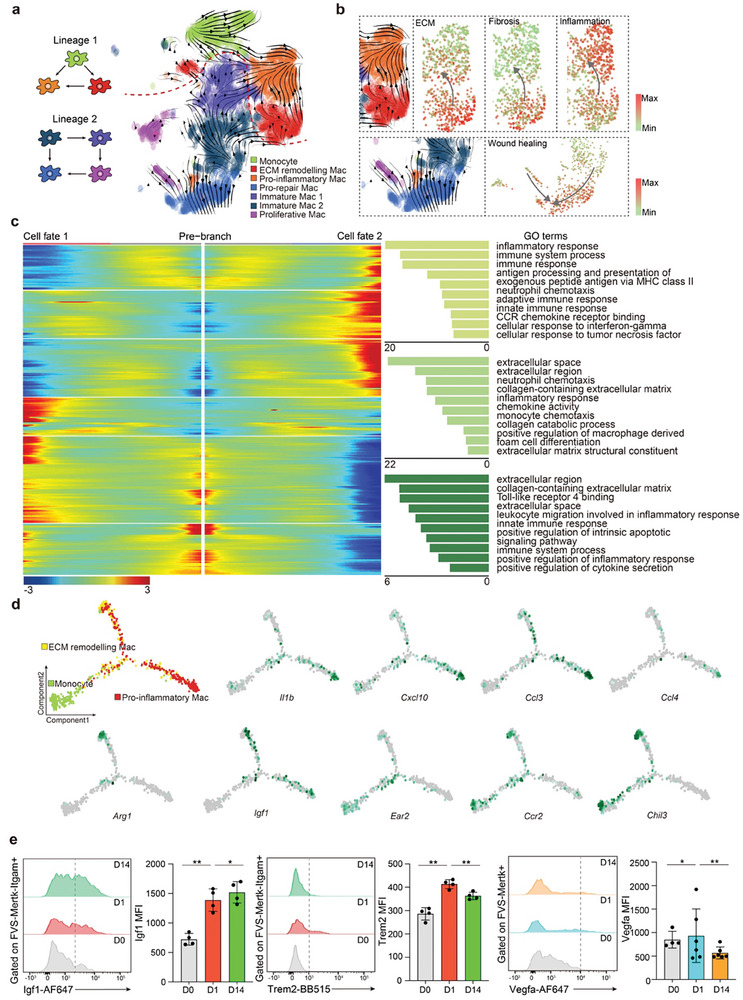
Macrophage trajectories during repair and fibrosis. a) RNA velocity analysis in each sub‐clusters. b) Inflammation, fibrosis, ECM, and wound healing scores along trajectory. c) Gene expression dynamics and Gene Ontology pathway enrichment analysis along pseudotime (using Monocle2). d) Pseudotime‐dependent gene expression along the lineage 1 and corresponding feature plots. e) Flow cytometric analysis of the expression of Itgam/Igf1, Itgam/Trem2, and Mertk/Vegfa in the injured kidneys at days 0, 1, and 14 post‐UIR.

To assess the potential origin and cellular differentiation of the mononuclear phagocytic system, we next defined cell lineage relationships using pseudotemporal cell ordering by RNA velocity analysis. We retrieved two distinct trajectories (**Figure** **5**a). Starting at 1 day post‐UIR, monocytes differentiated along lineage 1 toward EAMs, consistent with the high expression of *Ccr2*, *Retnla* and *Ear2* as markers of early activation of macrophages and typical monocyte‐recruiting marker (Figures 3a and 5d). Notably, this cluster differentiated into pro‐inflammatory Mac with the highest injury score at the endpoint of lineage 1 (Figure 5b,d). These analyses indicated a differentiation trajectory from EAMs to high‐inflammation and fibrosis Mac. Lineage 2 was composed of homeostatic tissue‐resident macrophages. We observed an alternative differentiation path from tissue resident cluster 4 toward pro‐repair Mac in UIR kidneys (Figure 5a). Diffusion mapping revealed the highest wound healing score at the endpoint of lineage 2 (Figure 5b). Notably, on day 1 post injury, part of resident cluster 4 transformed into proliferative Mac, indicative of a transitory differentiation state (Figure 5a). This highlighted that kidney injury induces local proliferation and self‐renewing of resident macrophages at the site of injury to regulate wound healing early after AKI.

Using Monocle2, we discovered differentially expressed genes along monocyte differentiation trajectories to further characterize EAMs. Module 1 represented upregulated genes during monocyte to pro‐inflammatory Mac differentiation (Figure 5c), which contained typical inflammatory markers such as *Il1b*, *Cxcl10*, *Ccl4*, and *Ccl3* at the endpoint of pro‐inflammatory Mac, and displayed ontology terms consistent with inflammatory response and immune system process (Figure 5d). Module 2 included a set of genes that were increased during monocyte‐to‐EAMs differentiation, which contained ECM components or molecules markers involved in ECM interaction and remodeling such as *Fn1*, *Arg1*, *Ctsd*, *Pf4*, *Lgals1*, and *Lgals3* (Figure 5c,d). These data identified the key signals involved in the process of monocyte differentiation that contributed to fibrosis. Further, the flow cytometry analysis of the phenotypic characteristics of these populations showed that the Itgam^+^ infiltrating macrophages expressed the pro‐fibrotic marker Igf1 and lipid metabolism marker Trem2. As the inflammation and fibrosis progressed in the UIR‐injured kidney, the expression of Igf1 reached its peak at 14 days. Moreover, we found that Mertk^+^ resident macrophages exhibited upregulation of Vegfa early at 1 day after UIR surgery, indicating that renal‐resident macrophages play a pro‐repair role such as promoting angiogenesis early after AKI (Figure 5e; Figure S4a–c, Supporting Information).

Collectively, these results support that two major cell lineages exist in the ischemic kidney. Renal resident macrophages differentiated into the pro‐repair Mac subsets as a repair response after injury, while EAMs were differentiated from monocytes which thereafter transitioned toward a pro‐inflammatory Mac and contributed to chronic inflammation and fibrosis. Distinct transcriptional programming models were observed that may determine the macrophage ontogeny.

### 2.6 Interaction of EAMs with Fibroblast

Finally, we sought to determine the cell communication network involving EAMs. Interestingly, kidneys of the sham group posited only limited interactions between endothelial cells and proximal tubule cells, while increasing cell‐cell communications were observed after UIR and especially in late fibrosis stages (**Figure** **6**a). We found that EAMs could directly interact with endothelial cells, fibroblasts, repairing PT, and neutrophils through the ligand‐receptor pairs such as Grn‐Sort1, Vsir‐Igsf11, Anxa1‐Fpr1, and Anxa1‐Fpr2, respectively (Figure 6b).

Figure 6Ligand–receptor interactome of communication between macrophages and other cells. a) Heatmap of the number of relevant ligand‐receptor interaction pairs predicted by CellChatDB between main kidney cell types in cell‐cell interaction study. Scale = number of interactions. b) Dot plot showing expression of ligand‐receptor pairs in EAMs and all other clusters in each time points. c) UMAP scRNA‐seq and heatmap of select ligands expressed by Mac sub‐clusters and cognate receptor expression by fibroblasts on days 14 and 28 post injury. d) Co‐expression pattern of *Igf1* and *Igf1r* in spatial transcriptomics dataset. Spatial feature plots showing the expression pattern of ligand gene *Igf1* (red spots), receptor gene *Igf1r* (blue spots), and co‐expression pattern (purple spots). e) Multicolour immunohistochemistry staining of Igf1^+^Cd68^+^ cells (Igf1 and Cd68) and Igf1r^+^α‐SMA^+^ cells (Igf1r and α‐SMA) in each injury time points. Scale bars, 10 µm. The heatmaps show the density of Igf1^+^ Cd68^+^ cells and Igf1r^+^ α‐SMA^+^ cells. Results of nearest neighbor distance used to compute areas in which Igf1^+^Cd68^+^ cells (blue) and Igf1r^+^α‐SMA^+^ cells (yellow) lie within ≈1 µm of each other. The box plots show the quantification results. n = 15. Ligand–receptor interactome of communication between macrophages and other cells. f) The representative images of IGF1 and CD68 immunofluorescence costaining in normal, AKI, and CKD kidneys and g) its correlation with fibrosis (Masson staining). h) The urinary sediment, supernatant, and serum IGF1 concentrations of healthy control (n = 15), patients with AKI (n = 15) and CKD including CKD 2–3 stage (n = 15) and CKD 3–5 stage (n= 15) and its correlation with GFR‐EPI. *P < 0.05. i) Western blots of Col1a1, a‐SMA, Vim, and PCNA after stimulation with IGF1 (IGF1 10 ng ml^−1^ 24 h and IGF1 10 ng/ml 48 h) in NRK49F cells. j) The representative images of IGF1 and CD68 immunofluorescence costaining in NRK49F cells after stimulation with IGF1 (IGF1 10 ng ml^−1^ 24 h and IGF1 10 ng ml^−1^ 48 h). k) Working model for spatiotemporal dynamics of macrophage heterogeneity in the process of AKI‐to‐CKD transition.

**Figure:**
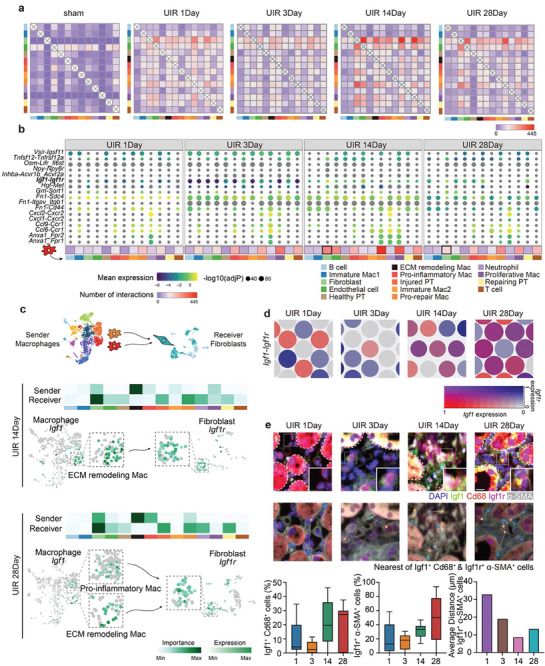

**Figure:**
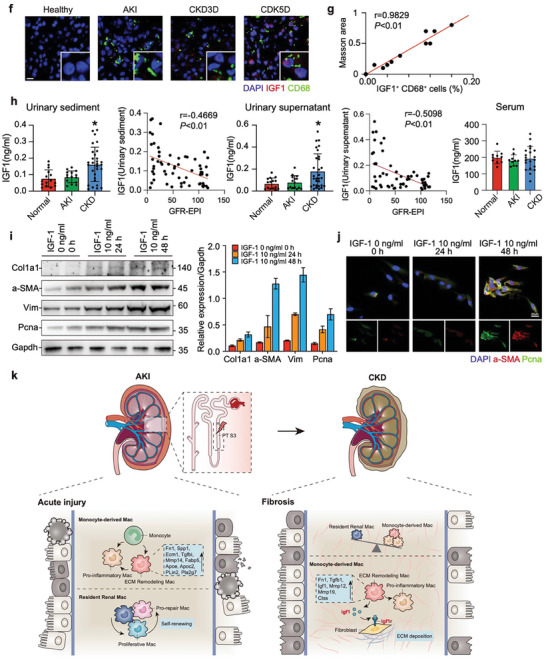

As revealed using CellChatDB that predicts the ligands from sender‐cells to interact with specific targets of receiver‐cells, several significant ligand‐receptor interactions were involved in ECM regulation. Igf1‐Igf1r, Fn1‐Cd44, Fn1‐Sdc4, Grn‐Sort1, and Tnfsf12‐Tnfrsf12a pairs were found between EAMs and fibroblasts (Figure 6b). We speculated that these pro‐fibrosis communication pairs, associated with pro‐fibrotic signals, are crucial in the activation of fibroblasts and regulation of ECM remodeling. Of these, we identified *Igf1* derived from EAMs as the top ligand that can interact with most of the fibroblast targets in the late phase of UIR (days 14 and 28) (Figure 6c).

To confirm the findings from the interactome, the expression of *Igf1*‐*Igf1r* pairs were validated in the spatial transcriptomic renal tissue slices. We found that *Igf1* and *Igf1r* were highly co‐expressed in niche cells of intra‐spots in late chronic stages (Figure 6d). Using multicolour immunohistochemistry (mIHC), we observed that Igf1^+^Cd68^+^ macrophages and Igf1r^+^α‐SMA^+^fibroblasts mainly localized in the tubulointerstitium which was particularly prominent in the chronic phase (Figure 6e; Figure S5a, Supporting Information). Importantly, the density and nearest analysis of HALO Image Analysis Platform revealed the physical proximity between Igf1^+^Cd68^+^ macrophages and Igf1r^+^α‐SMA^+^fibroblasts (Figure 6e; Figure S5b, Supporting Information), supporting their direct interaction. Additionally, we observed an increase in infiltrating Igf1^+^Cd68^+^ macrophages and Igf1r^+^α‐SMA^+^ fibroblasts, as well as a decrease in the distance between them, in the tubulointerstitium during chronic stages compared to the sections from the acute stages of kidney injury (Figure 6e). These results suggested the potential cell‐cell interactions between EAMs and fibroblasts. To further investigate the potential pathological significance of infiltrated IGF1^+^ macrophages in human AKI and CKD, we took advantage of an AKI and CKD cohort. Interestingly, we found the increasing expression of IGF1/CD68 co‐exist with the fibrosis area in kidney tissues of CKD patients (Figure 6f,g). Surprisingly, excretion of IGF1 in both urinary sediment and supernatant significantly increased in patients with CKD and was negatively correlated with GFR‐EPI (Figure 6h). To further verify the above finding link with fibrosis, we detect the influence of IGF1 on fibroblast cells using western blot and immunofluorescence. Impressively, the expression of Col1a1, a‐SMA, Vim and PCNA were substantially increased in fibroblast cell after IGF1 stimulation (Figure 6i,j). Thus, we proposed that IGF1^+^ macrophages could be a critical player in AKI to CKD transition.

## 3 Discussion

Chronic kidney disease (CKD) is a global public health problem.^[^ ^41^ ^]^ The transition of AKI to CKD is a key risk factor for the development and progression of CKD.^[^ ^42^ ^]^ During this process, unresolved inflammation and fibrosis represent the key event that promotes AKI to CKD transition, where macrophages served as a critical driver.^[^ ^8^, ^10^, ^43^ ^]^ However, the spatiotemporal dynamics of macrophages in this pathological process are poorly understood. Here we have delineated a comprehensive map of the macrophage populations during AKI to CKD transition in a mouse model of ischemic kidney injury by integrating spatial transcriptomics with single‐cell gene expression profiles.

The microenvironment and cellular communication are known to play fundamental roles in shaping the plasticity, function and localization of macrophages in different stages of kidney disease.^[^ ^44^, ^45^, ^46^, ^47^ ^]^ Previously, macrophages are classified as M1/M2 phenotypes in the progression of renal fibrosis. Here, we found that macrophage subsets changed dynamically in a more nuanced manner during the entire process of chronic progression following renal injury. Specifically, the proportions of the pro‐repair cluster dropped dramatically in late chronic stage while the pro‐inflammatory and ECM remodeling clusters increased continually. Stewart BJ et al. found that there was a cross‐talk between epithelial cells and immune cells that may determine the localization of macrophages and neutrophils to the infected regions of the kidney.^[^ ^48^ ^]^ However, the exact mechanism is largely unclear. In this study, by using integrative spatial and single‐cell analysis, we clearly demonstrated that macrophages are preferentially recruited to the injured S3 segment of the proximal tubular cells with the strong spatial dependency with neutrophils at the early stages but dependency with fibroblasts at chronic stages, suggesting that they may be involved in modulating the phenotype of renal macrophages in the vicinity of injured areas.

Although the macrophage heterogeneity is strongly shaped by the tissue environment with distinct lineages driving the functional characteristics, recent studies have indicated that monocyte‐derived macrophages recruited to the injured kidney and augment inflammatory responses, whereas tissue‐resident cells tend to mitigate inflammation and restore homeostasis.^[^ ^10^, ^44^, ^49^, ^50^ ^]^ However, the fate and dynamics of these cells are poorly understood. Our results suggested that renal resident macrophages exhibited remarkable proliferative characteristics after injury and differentiated into pro‐repair Mac from proliferative and immature status which contributes to tissue repair. In addition, we found ECM‐remodeling Mac was derived from monocytes and exhibited a phenotype shift toward pro‐inflammatory Mac thereafter, which may represent a new critical population associated with unresolved chronic inflammation and fibrosis. Interestingly, emerging data from multiple mouse models of nonalcoholic steatohepatitis (NASH) and idiopathic pulmonary fibrosis (IPF) have also shown significant role of infiltrating macrophages derived from monocytes in fibrotic niches and ECM remodeling activity.^[^ ^51^, ^52^, ^53^, ^54^, ^55^ ^]^ As such, the emergence of macrophages characterized with high levels of ECM related genes served as a critical player in organ fibrosis.

A notable aspect of the EAMs is its appearance early after the injury and persistence throughout the chronic phase with prominent function of ECM regulation among those diverse subsets of macrophages. Previous studies had suggested that macrophages played an essential role in regulating ECM remodeling via secretion of ECM components, production of ECM‐modulating proteases, and regulating the activation of ECM‐producing fibroblasts or macrophage‐to‐myofibroblast transition (MMT),^[^ ^56^, ^57^ ^]^ which could promote the survival and functional maintenance of macrophages. However, the precise function of this macrophage subset in the transition of AKI to CKD has not been fully clarified. We found that EAMs expressed high levels of lipid metabolism and phagocytosis genes in addition to ECM‐related component and proteases. This lipid metabolism signature is reminiscent of “Lipid‐Associated Macrophages” (LAM) found in the context of obesity, which highly express genes encoding ECM components and phagocytosis and promote fibrogenesis.^[^ ^51^, ^58^ ^]^ Macrophage scavenger receptor CD36 mediated the uptake of long‐chain fatty acids which activated fibrogenic signaling and promoted renal fibrosis.^[^ ^59^ ^]^ Our results strongly suggest that co‐regulation of ECM and lipid metabolic reprogramming represent a key aspect of EAMs biology during fibrogenesis.

Finally, our study provides a new perspective on the function of IGF‐mediated cellular communication in fibrogenesis. Excessive or sustained production of growth factors is a key mechanism of fibrosis.^[^ ^60^ ^]^ Here, we found that EAMs released exaggerated levels of growth factors Igf1 and Tgfb1, especially in the late fibrosis stages. Igf1 is a member of the small family of single‐chain polypeptides and have key roles in diabetes, inflammation, and fibrosis.^[^ ^61^ ^]^ Previous studies have suggested that Igf1 could activate fibroblasts and transformed to the myofibroblast which is involved in renal fibrosis.^[^ ^62^ ^]^ However, the role of macrophages‐derived IGF1 in renal fibrosis warranted elucidation. In the current study, we found that *Igf1* is predominantly expressed in EAMs, while *Igf1r* is mainly expressed in epithelial cells and fibroblasts. Furthermore, during the fibrotic phase, there is a significant increase in the expression level of IGF1R in fibroblasts. Notably, EAMs had a strong interaction with fibroblasts through the Igf1‐Igf1r signaling axis and IGF1 stimulated fibroblast activation and growth, suggesting its critical role in mediating the cellular interaction between macrophages and fibroblast in renal fibrosis. Impressively, the presence of kidney IGF1/CD68^+^ macrophage infiltration and the relevance of renal IGF1/CD68 expression to the degree of renal fibrosis were confirmed in human CKD. The urinary excretion of IGF1 in CKD patients also correlated with the severity of kidney fibrosis. Taken together, these data suggested that IGF1 might also serve as a potential prognostic and novel predictive biomarker for fibrosis.

There are several limitations in our study. First, we combined three mice in each time point for analysis. While each of these mice has its individual differences, the protocol of creating UIR model is robust and reproducible. In the future, sample multiplexing approaches will address this limitation. Second, UIR is a unique injury model in which the innate immune response and interaction with the adaptive immune system are involved. Combining data sets of other models in the atlas might prove beneficial in the future. Meanwhile, validation specific cell types in human datasets is in urgent need for identification of their consistency across different species. Additionally, although we stimulated fibroblasts and promoted their activation using IGF1 in vitro, in vivo inhibition of IGF will be needed to evaluate whether the transition from AKI to CKD is prevented.

In conclusion, the present study demonstrated the spatiotemporal dynamics of macrophage heterogeneity in the process of AKI to CKD transition. Renal resident macrophages differentiated into the pro‐repair Mac subsets, while EAMs originated from monocytes persistently present and transformed toward a pro‐inflammatory Mac which contributed to chronic inflammation and fibrosis. Our findings provided a new insight about the diverse role of macrophage during AKI to CKD transition, which will provide a novel therapeutic strategy to prevent renal fibrosis after acute kidney injury.

## 4 Experimental Section

### Renal Unilateral Ischemic Reperfusion Injury Model

The trials used C57BL/6 male mice (6‐8‐week‐old) acquired from Beijing Vital River Laboratory Animal Technology Co., Ltd., Beijing, China. Mice were anesthetized with isoflurane treated to ischemia by clamping the right renal pedicles with non‐traumatic microaneurysm clamps for 35 min, and then reperfused by releasing the clamps. The mice were kept at a constant body temperature of 37 °C. Mice were euthanized at day 1, day 3, day 14, or day 28 after UIR. The same surgical procedures were performed on sham mice, except that the renal pedicles were not constricted. At the end of the experiment, the mice were euthanized by inhaling carbon dioxide after isoflurane anesthesia, and blood and kidney tissues were collected. Blood and urine were used for analysis of renal function and renal injury biomarkers, while organs were used for observation of tissue lesions and relevant indicator tests.

### Patient Samples

The study was approved by the Ethical Committee of Zhong Da Hospital (approval number: 2017ZDSYLL107‐Y02), Southeast University and the informed consent were obtained from all participants. Healthy controls (n = 15) and patients with AKI (n = 15), CKD 2–3 stage (n = 15) and CKD 3–5 stage (n = 15) were enrolled in this study. The clinical characteristics of all patients are summarized in **Table** **2**. Renal biopsy was used for IGF1 and CD68 immunostaining. The concentrations of IGF1 in the plasma and urine samples from the renal biopsy cohort were measured by enzyme‐linked immunosorbent assay (ELISA) using human IGF1 Quantikine kit (Cloud‐Clone, China) according to the manufacturer's instructions. Plasma and urine samples for the healthy controls were from 15 ethically matched volunteers.

**Table 2:** Clinical characteristics of all patients.

Number	GFR‐EPI	IGF1‐sediment(ng/ml)	IGF1‐supernatant(ng/ml)	CKD stage	Age	Gender	Collection Date	Total protein	Albumin	BUN	Scr(umol/l)	TG	TC	GLU	Pro	Urine Protein(g/l)	24UTP	ACR
1	41.15	0.135	0.196	CKD 3	71	Male	2023/9/25	63.9	38.4	8.4	127	0.67	3.54	4.22	0	–	–	–
2	65.9	0.019	0.052	CKD 2	64	Female	2023/9/25	47	26.8	11	82	1.68	8.54	4.55	2	3.78	6.804	3188.2
3	108.13	0.035	0.019	CKD 2	61	Male	2023/9/23	43.5	25.1	3.3	70	0.89	6.01	4.18	2	1.43	1.144	–
4	65.94	0.035	0.055	CKD 3	59	Male	2023/10/8	68.7	44.8	7.4	106	1.7	5.87	8.4	0	0.08	0.136	–
5	47.6	0.017	0.019	CKD 3	60	Male	2023/10/7	41.4	19.5	15.5	138	1.15	4.53	4.37	3	2.47	4.446	3524.9
6	75.44	0.065	0.113	CKD 2	68	Male	2023/10/8	57.5	37.8	11.4	90	1.99	5.11	5.79	0	0.09	0.18	80.76
7	86.4	0.102	0.147	CKD 2	75	Female	2023/10/7	49	33.8	8.1	59	2.52	4.07	5.1	0	0.28	0.532	214.78
8	81.46	0.023	0.114	CKD 2	59	Male	2023/10/7	35.6	21.3	14.7	89	1.07	4.9	3.71	4	4.48	7.616	134.53
9	110.66	0.198	0.046	CKD 1	53	Female	2019‐05‐13	47.4	22.7	3.6	44	4.43	9.74	5.14	2	0.395	0.592	1674
10	77.31	0.172	0.093	CKD 2	77	Female	2021‐04‐08	63	35.9	7.1	66	5.47	5.96	9.38	1	0.298	0.775	1818.5
11	60.8	0.166	0.052	CKD 2	72	Male	2019‐07‐03	71.7	43.5	8.4	105	2.23	2.45	7.49	0	0.156	0.312	137.08
12	44.28	0.181	0.042	CKD 3	49	Male	2019‐06‐28	64.5	40.9	9.8	156	2.48	4.32	9.54	0	0.081	0.182	7.15
13	73.69	0.174	0.089	CKD 2	43	Male	2019‐05‐30	76.9	44.3	5.4	106	2.17	3.51	5.09	0	0.109	1.164	85.22
14	92.64	0.161	0.119	CKD 1	63	Female	2019‐03‐06	43	23.2	3.1	61	2.51	7.61	5.51	3	2.25	2.7	177.22
15	115.57	0.079	0.137	CKD 1	39	Female	2019‐01‐07	74.6	44	6.2	52	1.12	5	4.64	2	0.432	0.518	212.42
16	52.39	0.114	0.148	AKI	54	Male	2023/9/20	66.3	39.5	15.5	132	1.28	4.14	5.86	0	–	–	10.07
17	8.3	0.037	0.029	AKI	67	Male	2023/9/22	64.4	44	18.8	562	2.05	4.72	4.74	0	0.35	0.84	92.47
18	71.5	0.04	0.035	AKI	62	Male	2023/10/10	53.7	35.6	10.9	112	2.19	5.4	5.44	1	0.26	0.676	259
19	10.02	0.097	0.111	AKI	79	Male	2023/10/26	62.1	35.7	21	355	1.39	4.81	4.37	0	0.62	0.558	>296.18
20	49.4	0.087	0.021	AKI	25	Male	2023/11/16	78.1	52	7.7	164	1.94	4.8	4.8	0	–	–	14.74
21	12.6	0.053	0.018	AKI	27	Male	2023/10/26	60.3	31.6	24.2	502	3.02	4.3	4.34	3	0.17	0.391	–
22	60.3	0.133	0.027	AKI	53	Male	2020‐10‐	61.8	40.5	7.3	118	1.04	4.16	4.94	Trace	0.117	0.176	141.97
23	61.5	0.064	0.112	AKI	36	Male	2019‐12‐	46.6	23.7	11.2	129	5.52	7.83	6.14	3	18.31	23.803	161.97
24	12.5	0.084	0.213	AKI	35	Male	2019‐2‐	60.5	37.6	16.7	481	1.46	4.11	4.76	1	0.253	0.557	162.5
25	35.8	0.083	0.037	AKI	40	Male	2019‐2‐	45.7	21.5	12.8	196	2.63	13.01	5.28	3	20.679	16.543	–
26	12.13	0.112	0.076	AKI	75	Male	2023‐10‐	47.1	24.5	38.6	394	1.91	3.45	13.8	1	1.03	1.03	506.83
27	37.95	0.031	0.031	AKI	69	Female	2023‐10‐	56.9	33.5	13.6	125	2	2.99	5.46	0	0.34	0.442	23.73
28	52.86	0.123	0.093	AKI	58	Male	2023‐9‐	61.8	41.7	4.7	128	4.4	4.67	4.55	0	–	–	10.89
29	33.36	0.098	0.127	AKI	62	Male	2023‐6‐	64.1	38.9	12.8	183	1.35	2.3	3.62	0	0.03	0.03	6.79
30	26.27	0.144	0.136	AKI	86	Male	2023‐6‐	71.9	37.4	29.5	194	1.81	3.09	4.52	0	0.08	0.32	28.16
31	5.58	0.013	0.032	CKD 5	76	Female	45 193	58.2	32.1	19.4	586	1.58	1.55	4.43	2	–	–	–
32	6.82	0.365	0.458	CKD 5	37	Male	45 209	56.9	37.1	18.1	787	1.46	3.14	3.93	0	0.7	0.98	–
33	10.12	0.16	0.211	CKD 5	70	Female	45 206	56.3	32.6	24.3	371	2.46	4.5	8.85	2	2.44	4.392	–
34	7.7	0.238	0.299	CKD 5	67	Female	45 206	71.7	42.6	14.6	473	1.86	4.27	5.21	1	–	–	–
35	9.88	0.291	0.397	CKD 5	74	Male	45 209	51	27	21.5	467	1.17	2.77	4.54	1	–	–	563.35
36	20.34	0.149	0.114	CKD 4	55	Male	45 193	67	40	13.7	287	0.88	2.21	4.51	±	–	–	199.94
37	9.65	0.371	0.541	CKD 5	74	Male	45 225	62.8	38.2	24.1	476	1.14	3.31	3.91	0	0.16	0.208	46.91
38	28.26	0.134	0.411	CKD 4	41	Female	2018‐04‐11	66.8	36.1	6	187	1.55	3.82	4.62	3	1.619	3.886	–
39	22.72	0.176	0.373	CKD 4	67	Male	2018‐04‐13	50.9	25.6	20.6	244	1.33	3.29	4.51	1	0.568	1.136	–
40	13.01	0.243	0.462	CKD 5	73	Female	2018‐05‐21	61.9	27.1	22.9	295	2.48	4.95	6.15	2	1.808	2.17	–
41	5.26	0.299	0.291	CKD 5	40	Female	2018‐05‐29	47.2	23.8	24.8	756	2.39	5.43	3.23	3	1672	0.334	1449.9
42	25.45	0.345	0.313	CKD 4	66	Female	2023‐11‐	44.4	24	6.6	177	0.75	3.56	4.01	3	4.76	3.332	–
43	22.9	0.198	0.031	CKD 4	46	Female	2023‐11‐	63.3	36.8	10.2	217	1.09	4.88	4.14	3	1.4	3.36	1459
44	21.27	0.221	0.045	CKD 4	78	Male	2023‐10‐	50.6	28.6	12.8	242	0.78	3.11	3.81	0	0.51	1.071	376.23
45	12.53	0.124	0.211	CKD 5	88	Female	2023‐10‐	40	21.2	3.6	280	1.07	3.79	4	3	0.02	0.024	8055.2
46	107.42	0.021	0.031	Healthy	25	Female	2023/8/26											
47	101.23	0.032	0.022	Healthy	28	Male	2023/8/26											
48	114.31	0.121	0.113	Healthy	22	Male	2023/8/26											
49	104.22	0.093	0.087	Healthy	35	Male	2023/8/26											
50	117.21	0.168	0.031	Healthy	32	Male	2023/8/26											
51	102.21	0.127	0.089	Healthy	29	Female	2023/8/27											
52	108.44	0.018	0.066	Healthy	30	Male	2023/8/27											
53	117.23	0.012	0.033	Healthy	43	Male	2023/8/27											
54	114.76	0.021	0.031	Healthy	57	Female	2023/8/27											
55	102.57	0.034	0.012	Healthy	32	Female	2023/8/27											
56	103.91	0.111	0.064	Healthy	22	Female	2023/8/27											
57	106.37	0.092	0.092	Healthy	47	Female	2023/10/17											
58	102.96	0.081	0.084	Healthy	60	Male	2023/10/17											
59	106.22	0.083	0.044	Healthy	51	Female	2023/10/17											
60	109.54	0.024	0.051	Healthy	31	Male	2023/10/17											

### Morphological Studies

A normal process was used to obtain formalin‐fixed, paraffin‐embedded mouse kidney slices (4‐m thickness). A standard protocol was followed for hematoxylin and eosin (H&E), Periodic Acid‐Schiff (PAS), and masson staining. A conventional protocol for immunofluorescence staining was followed.

### Single‐Cell Suspension Preparation

Each sample included three mice. Anesthetized mice were perfused with pre‐chilled 1 × PBS via the left cardiac ventricle. Samples were minced into 1 mm^3^ cubes before being digested with the Multi Tissue dissociation kit (Miltenyi, 130‐110‐203). Using 21 G and 26 1/2 G syringes, the tissue was homogenized. In 3 ml of 1640 medium (Gibco, USA), 0.25 g of tissue was digested with 1350 µl of collagenase I, 375 µl of collagenase IV, and 180 µl of hyaluronidase and incubated for 40 min at 37 °C. 10% FBS inhibited the response. The solution was then filtered using a 70 µm cell strainer. After centrifugation at 400 g for 5 min, the cell pellet was treated on ice for 5 min with 1 ml of RBC lysis solution (Miltenyi, 130‐094‐183). Countstar (Alit Biotech, Rigel S2) was used to determine cell number and viability. This approach produced a single‐cell suspension with a viability of more than 90%.

### Cell Culture and Cell Treatment

The fibroblastic clone of NRK (mixed culture of normal rat kidney) cell line (NRK49F cell) was obtained from the American Type Culture Collection (Manassas, VA) and cultured in DMEM/F12 with 10% FBS in a 37 °C incubator with 5% CO_2_. Cells were pretreated with 10 ng mL^−1^ IGF1 (UA BIO, UA040099) for 24/48 h. Some cells were detected by immunofluorescence and WB analysis.

### Western Blot Analysis

Nearly 15 mg of total kidney tissue was homogenized in SDS lysis buffer, sonicated, and heated at 95 °C. Lysates were cleared by centrifuging (15 000 × g at 4 °C for 15 min). 15 µL of total lysate was loaded onto 11% SDS‐PAGE and subjected to electrophoresis (140 V, room temperature). Proteins were transferred onto PVDF membranes at 100 V on ice for 1 h. Membranes were incubated in 5% bovine standard solution (BSA) prepared in Tris‐buffered saline containing Tween‐20 (TBST) for 1 h at room temperature on an orbital rocker. Membranes were probed with: anti‐Col1a1 (dilution 1:1000; ab270993; Abcam), anti‐aSMA (dilution 1:1000; ab7817; Abcam), anti‐Vim (dilution 1:2500; ab92547; Abcam), anti‐PCNA (dilution 1:1000; ab29; Abcam). After primary antibody incubation blots were washed three times with TBST, HRP‐conjugated secondary antibodies (#7074, CST, dilution 1:2000) were probed for 1 h at room temperature prepared in TBST. Finally, blots were washed with TBST for 5 min each at room temperature. After applying the ECL color reagent and performing dark chamber exposure imaging, the gray value of the images was analyzed using ImageJ software v1.8.0 (NIH) and The final relative quantification values are the ratio of net band to net loading control. GraphPad Prism 9 was used to obtain statistical figures.

### Immunofluorescence Staining

Immunofluorescence analysis was performed on kidney sections (2‐µm thickness) and NRK49F cells. Slides were incubated with antibodies against anti‐aSMA (dilution 1:1000; ab7817; Abcam), anti‐CD68 (1:200, GB113109, Servicebio), anti‐PCNA (dilution 1:1000; ab29; Abcam). and incubated with secondary antibodies (ab150114 and ab150077, Abcam). Nuclei were stained with DAPI (Sigma–Aldrich) according to the manufacturer's instructions. Under a confocal microscope (FV3000, Olympus), 10 fields of view were randomly assigned, and the number of CDK12‐positive cells was counted in a blinded manner.

### scRNA‐Seq by 10 × Genomics

The cell suspension was fed onto the Chromium single cell controller (10x Genomics, GCG‐SR‐1) to form single‐cell gel beads in the emulsion according to the manufacturer's procedure using the Single Cell G Chip Kit (10x Genomics, 1 000 120). Reverse transcription was carried out on an S1000TM Touch Thermal Cycler (Bio Rad) at 53 °C for 45 min, 85 °C for 5 min, and 4 °C hold. The cDNA was produced, amplified, and tested for quality using an Agilent 4200 (done by CapitalBio Technology, Beijing). Single Cell 3′ Library and Gel Bead Kit V3.1 were used to create single‐cell RNA‐seq libraries. The libraries were finally sequenced on an Illumina Novaseq 6000 sequencer with at least 100 000 reads per cell and pair‐end 150 bp (sPtrEa1t5e0gy) (done by CapitalBio Technology, Beijing).

### Analysis of Single‐Cell RNA‐seq Data

*Alignment and quality control*: CellRanger was used to quantify raw fastq files that were aligned to the 10 mm (Ensembl GRCm38.93) reference genome. Seurat was used to control data quality, preprocess data, and do dimensional reduction analysis. Each time point is a pool of kidney from 3 mice. Following the compilation of gene‐cell data matrices from n = 12 UIR samples and n = 3 control samples, all 15 matrices were pooled, and poor‐quality cells with 200 expressed genes and mitochondrial gene percentages greater than 25 were eliminated. The remaining high‐quality cell barcodes were exported.

*Dimension‐Reduction and Cell Clustering*: The entire Seurat process was run again on the remaining 60010 high quality single cells to generate the final dataset used for all downstream analysis. The top 2000 highly variable genes were retrieved from each sample using the FindVariableFeatures function and then analyzed using principal component analysis (PCA). The main cell types were identified with the standard Seurat software at a resolution of 0.6. The data with a resolution of 0.6 were chosen for the target cell sub‐clustering analysis to demonstrate changes in the subtypes in each sample. Then, Seurat's RunUMAP function with dimension settings (1:30) was used for 2D visualization.

*Identification of marker genes and DEGs*: The FindAllMarkers function in Seurat was used to identify DEGs in cell clusters, and a list of marker genes was used for manual annotation of main cell types to the 13 found clusters in the final dataset. The heatmaps, and dot plots for the cell‐specific markers were generated using the Seurat DoHeatmap/DotPlot function.

*Enrichment analysis*: KOBAS software was used to perform GO and KEGG pathway enrichment with Benjamini‐Hochberg multiple testing adjustment. The R package was used to visualize the results.

*Monocyte/Macrophage sub‐clustering analysis*: The whole Seurat pipeline was run again, but this time just the barcodes of cells tagged as Mac/DC cells were used. The pipeline was run with the same settings as described before, giving 11 subclusters, including 7 macrophage subclusters. The subclusters were manually annotated using canonical marker genes and known functions, as detailed in previously published reports.

*The scoring and comparing of gene sets in scRNA‐seq data*: Gene sets comprising relevant markers were created based on previously published data on various macrophage lineages and phenotypic functions (Table 1). The gene set scores of each cell were produced using the Seurat package's “AddModuleScore” function and the “CellCycleScoring” function.

### scRNA‐seq Trajectory Analysis

*Monocle2*: Pseudotime trajectories were validated using the same cells as input using the Monocle packages. The differentialGeneTest function of Monocle2 with *q* < 0.01 was used to identify highly variable genes along pseudotime. The BEAM and plot_genes_branched_heatmap functions were used to evaluate individual branches.

*RNA velocity*: The Python‐based Velocyto command‐line tool and the Velocyto. R package were used as directed to compute RNA velocity. Using spliced and unspliced data, we used Velocyto to calculate the single‐cell trajectory/directionality. The SeuratWrappers package was used to load this subset into R. RNA velocity was calculated using a gene‐relative model with kNN cell pooling (k = 25). When viewing RNA velocity on the UMAP embedding, the parameter n was set at 200.

*Ligand–receptor interactions*: The CellChat library to predict cellular communication networks from single‐cell RNA seq data to examine cellular cross‐talk between different cell types was used.^[^ ^63^ ^]^ The Python software CellChat with database v1.1.3 to predict ligand‐receptor interactions was used. Only receptors and ligands expressed in >5% of the cells were considered.

*Statistics & reproducibility*: Unless otherwise specified, data were expressed as means ± SEM unless otherwise stated. Statistical analyses were indicated in the respective “Methods” section and Figure legends. According to the normalcy distribution, appropriate parametric or non‐parametric tests were performed. *P* < 0.05 was regarded as statistically significant. To establish sample size, no statistical procedure was applied. There were no data removed from the analyses.

### Spatial Transcriptome Sequencing

Cryosections of 10‐mm thickness were put on the Thermocycler Adaptor with the active surface facing up for 1 min at 37 °C, then fixed for 30 min in −20 °C with methyl alcohol before staining with H&E.

The Visum spatial gene expression slide and Reagent Kit (10x Genomics, PN‐1000184) were used to process the Visum spatial gene expression. 70 µl Permeabilization enzyme were added and incubated for 30 min at 37 °C. Each well was rinsed with 100l SSC before adding 75 µl reverse transcription Master Mix for cDNA synthesis.

RT Master Mix from the wells at the end of first‐strand synthesis was removed and added 75 µl 0.08 M KOH for 5 min at room temperature before removing the KOH from the wells and washing with 100 ul EB buffer. For second‐strand synthesis, 75 µl Second Strand Mix to each well was added. The cDNA amplication was carried out on a Bio Rad S1000TM Touch Thermal Cycler. Visum spatial libraries were created using the Visum spatial Library construction kit (10x Genomics, PN‐1000184). The libraries were finally sequenced using an Illumina Novaseq6000 sequencer with at least 100 000 reads per spot and a pair‐end 150 bp (PE150) reading method (done by CapitalBio Technology, Beijing).

### Spatial Transcriptome Sequencing Data Analysis

The ST‐seq data was bioinformatically processed using the R package Seurat (version 3.2.0). In brief, default values were used for normalization (“SCTransform”), dimensionality reduction (“RunPCA”), graph‐based clustering (“FindNeighbors” and “FindClusters” algorithms), UMAP visualization, and DEGs analysis. Cell2location with the default parameters to determine the cell‐type composition of each spot was used.^[^ ^64^ ^]^ The model basis was initialized using all markers, and unit variance normalization was performed.

MistyR (v1.2.1) was used to investigate the relationships between tissue organization and the spatial distribution of macrophages and other cells. The abundances of cell types determined with cell2location were used as predictors. To account only for the impacts of cell state activation, predictor cell state scores were masked to 0 whenever their score was less than 0.

### mIHC

All the Formalin‐fixed and paraffin‐embedded (FFPE) tissues used within the experimental operation were sectioned as slides of 4 µm thickness. The slides were deparaffinized in xylene for 30 min and rehydrated in absolute ethyl alcohol for 5 min (twice), 95% ethyl alcohol for 5 min, and 75% ethyl alcohol for 2 min sequentially. Wash the slides with distilled water 3 times. A microwave oven was used for heat‐induced epitope retrieval, and during epitope retrieval, the slides were immersed in boiling EDTA buffer (ZLI‐9079, zsbio, Beijing, China) for 15 min. Antibody Diluent/Block from Alpha X Bio was used for blocking. The mIHC experiments were performed by AlphaXPainter X30 (Alpha X Bio, Beijing, China). Primary antibodies for anti‐Igf1 (1:500, Abcam), anti‐Igf1r (1:200, Abcam), anti‐α‐SMA (1:500, Abcam), and anti‐CD68 (1:200, GB113109, Servicebio) were utilized on mice kidneys. 2–3 experienced pathologists assessed each part. All the primary antibodies were incubated for 1 h at 37 °C. Then slides were incubated with Alpha X Ploymer HRP Ms+Rb (Alpha X Bio, Beijing, China) for 10 min at 37 °C. Alpha X 7‐Color IHC Kit (AXT37100031, Alpha X Bio, Beijing, China) was used for visualization. After each staining cycle, heat‐induced epitope retrieval was performed to remove all the antibodies including primary & secondary antibodies. The slides were counter‐stained with DAPI for 5 min and enclosed in Antifade Mounting Medium (I0052; NobleRyder, Beijing, China). ZEISS AXIOSCAN 7 was used to scan multispectral pictures. Halo (v.3.4; Indica Labs) or QuPath (v.0.2.0) were used to count cells of interest.

## Conflict of Interest

The authors declare no conflict of interest.

## Author Contributions

Y.‐L.Z., T.‐T.T., and B.W. contributed equally to this work. Y.‐L.Z., T.‐T.T., and B.W. conceptualized and designed the experiments, interpreted experimental results, and wrote the manuscript, with contributions from all authors; Y.‐L.Z. conducted most of the experiments, with help from Y.W., Y.F., Q.Y., W.J., Y.Z., Z.‐L.L., M.W., Q.‐L.W., and J.S.; W.J. carried out animal experiments; S.D.C., H.‐Y.L., L.‐L.L., and B.‐C.L. conceptualized the study, provided supervision, interpreted all results, and wrote the manuscript. All authors read and approved the final paper.

## Supporting information

Supporting Information

## Data Availability

The data that support the findings of this study are available from the corresponding author upon reasonable request.
