# Supplementary material for: Identification of a Novel ECM Remodeling Macrophage Subset in AKI to CKD Transition by Integrative Spatial and Single‐Cell Analysis
Source: Adv Sci (Weinh). 2024 Aug 9;11(38):2309752. doi: 10.1002/advs.202309752 (PMC11481374; doi:10.1002/advs.202309752)
Supplement: Supplementary file 1 — Supporting Information [file ADVS-11-2309752-s001.docx]

**Supporting Information**


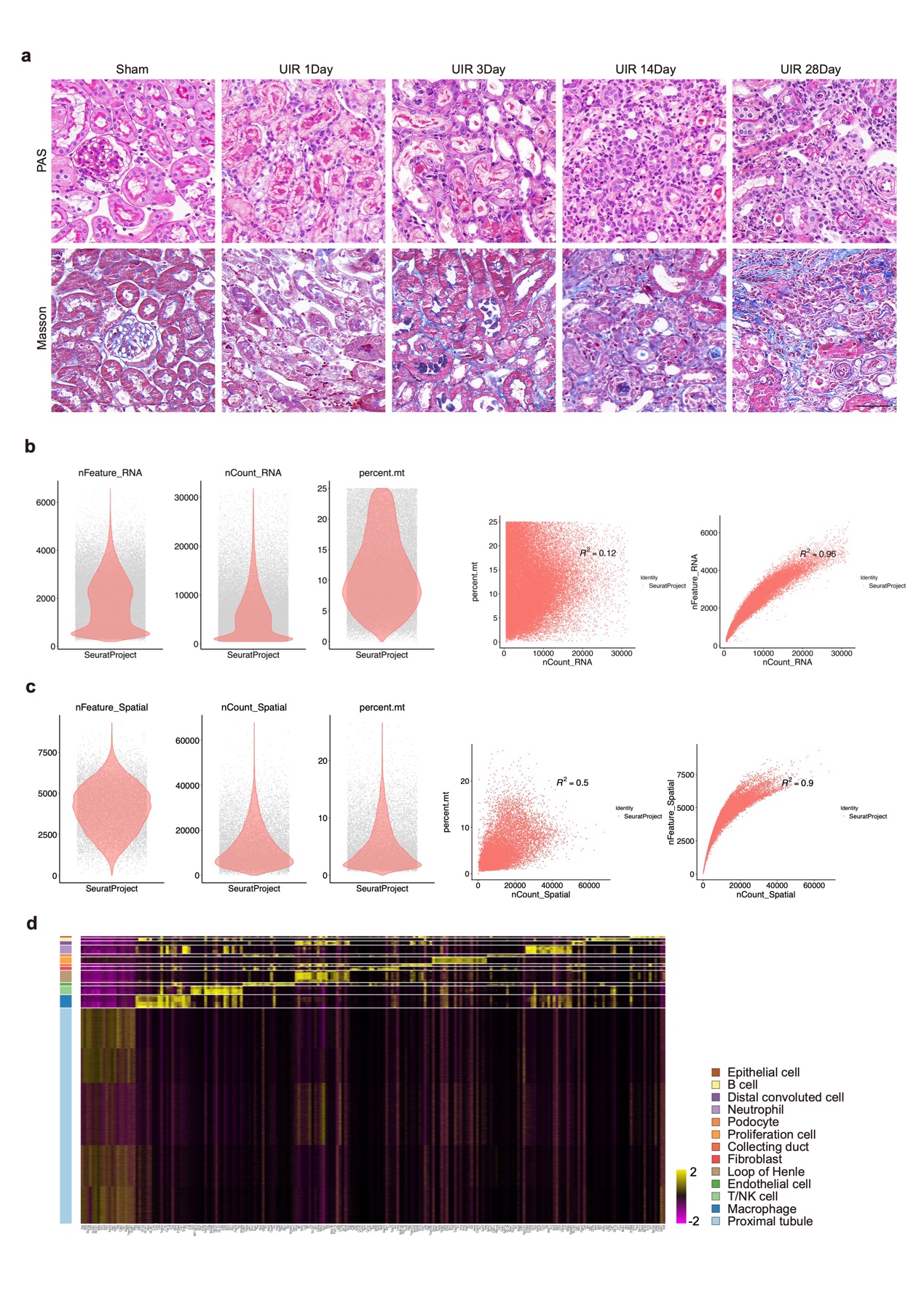


**Fig. S1| Single-cell analysis of renal tissue from unilateral ischemia-reperfusion injury mice.** Related to Fig. 1 **a**, Representative images of PAS stained (top) and Masson staining (bottom) at each timepoint. Scale bars, 50 μm. **b**, In kidney scRNA-seq datasets from all samples, the number of unique genes discovered per cell (left), the number of unique transcripts per cell (middle), and the proportion of mitochondrial transcripts (right) were all calculated. **c**, Number of unique genes discovered per cell (left), the number of unique transcripts per cell (middle), and the proportion of mitochondrial transcripts (right) in kidney spatial transcriptomics datasets from all samples. **d**, A heat map depicting the expression of the top 20 upregulated genes across cell types.

**
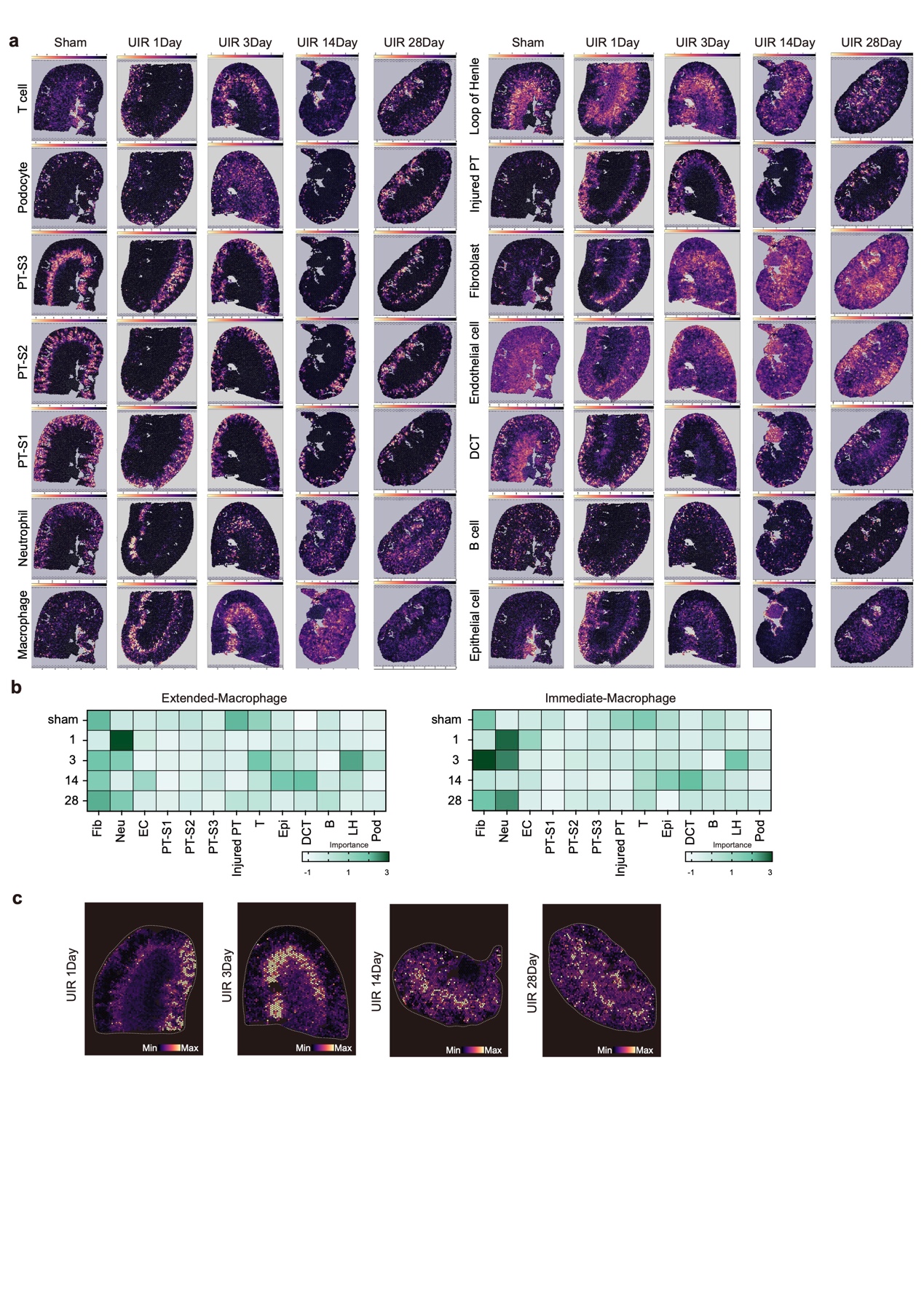
Fig. S2| Spatial transcriptomics data analysis.** Related to Fig. 2 **a**, Cell2location estimates the abundance of all cell types. **b,** Median relevance of cell-type abundances in predicting other cell types in the extended neighbourhood (left) and the immediate neighbourhood (effective radius of 15 spots) (right), as determined from spatially contextualized models. **c**, Cell2location cell-type abundance estimations of EAMs.

**
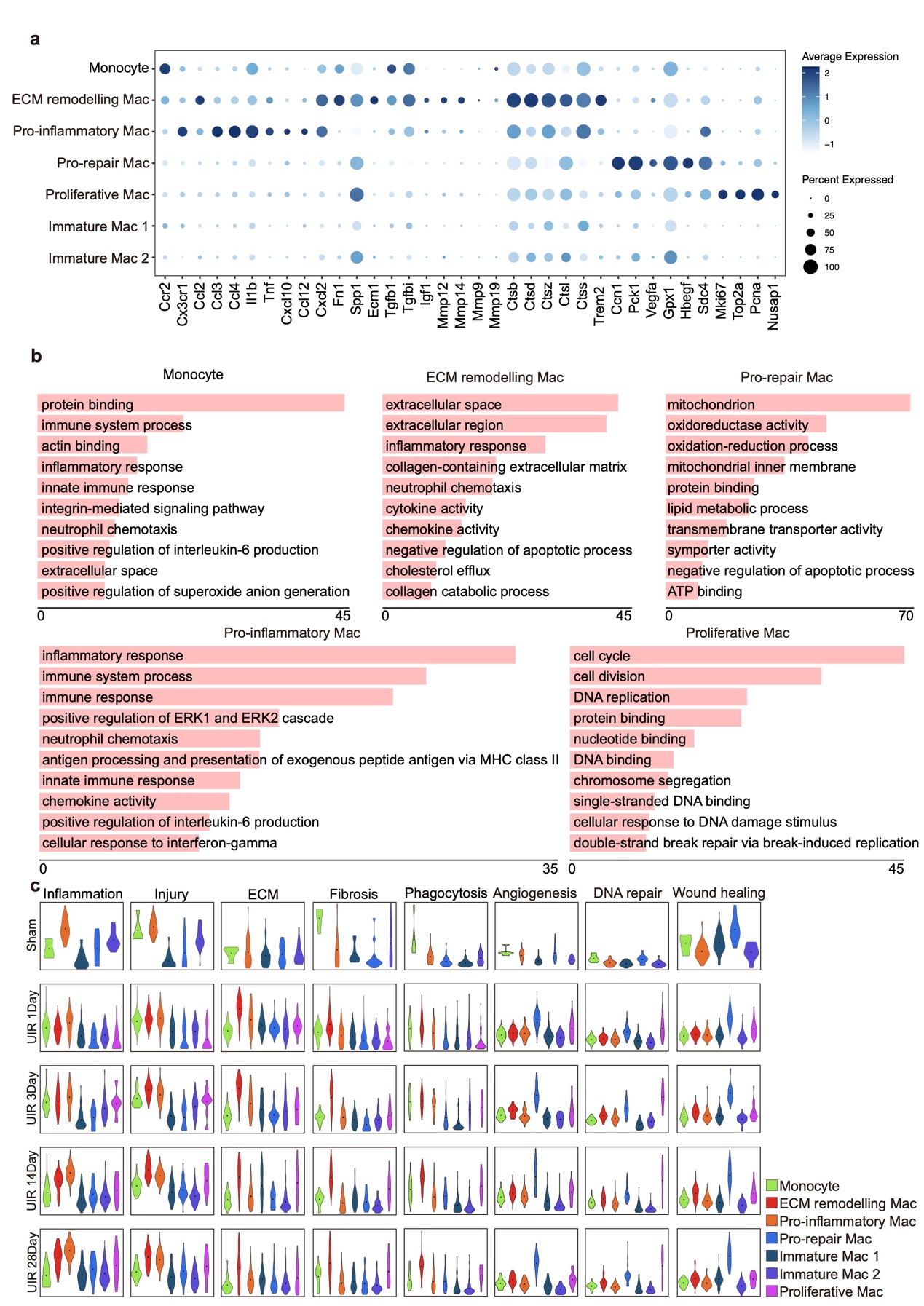
Fig. S3| Characterisation of renal monocytes/macrophages.** Related to Fig. 3 **a**, Dot plot showing expression of marker genes. **b,** Gene Ontology terms enriched from the differentially expressed genes of each monocyte/macrophage subsets compared to all other Mac clusters. **c**, Inflammation, injury, ECM, fibrosis, phagocytosis, angiogenesis, DNA repair, and wound healing scores of each monocyte/macrophage subsets in each time points.


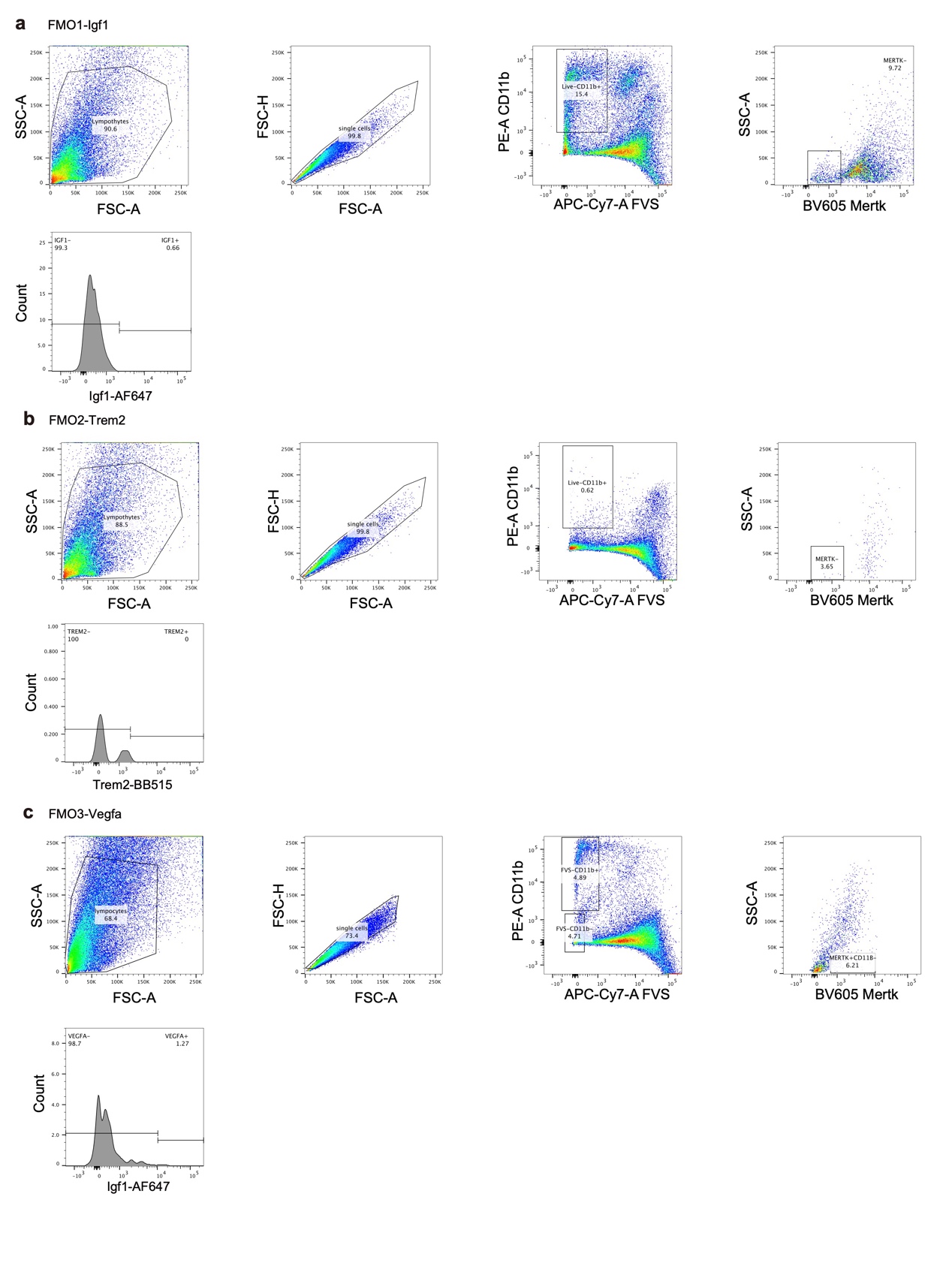


**Fig. S4| Characterisation of macrophages derived from different lineages.** Related to Fig. 5 **a**, Frequency of infiltrating Itgam^+^Igf1^+^ macrophages and **b**, Itgam^+^Trem2^+^ macrophages analyzed with flow cytometry. c, Frequency of resident Mertk^+^Vegfa^+^ macrophages.


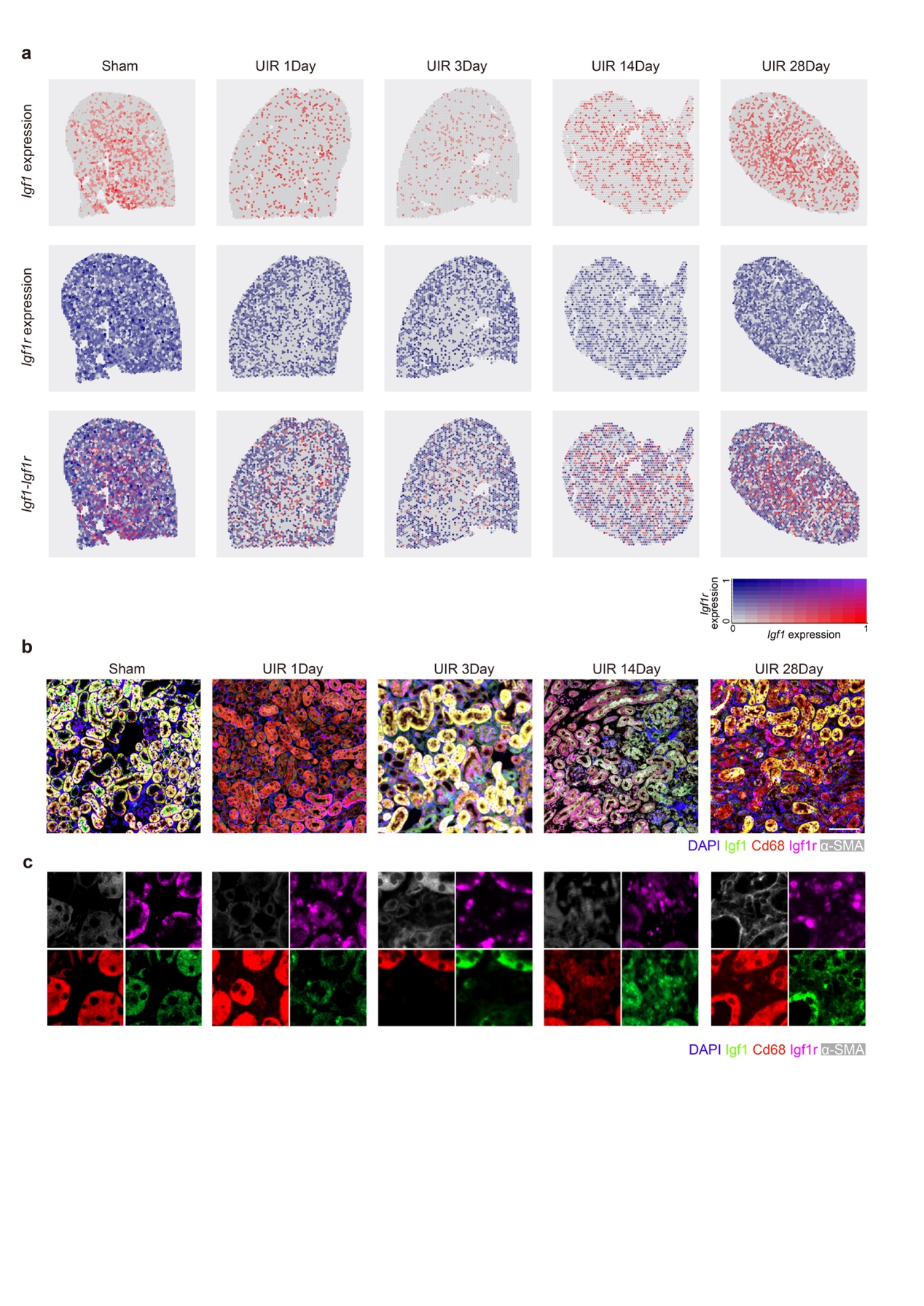


**Fig. S5| Characterisation of Igf1-Igf1r signaling axis.** Related to Fig. 6 **a**, Spatial feature plots showing the expression pattern of ligand gene *Igf1* (red spots), receptor gene *Igf1r* (blue spots), and co-expression pattern (purple spots) in each time points. **b**. Staining of Igf1^+^Cd68^+^ cells (Igf1 and Cd68) and Igf1r^+^α-SMA^+^ cells (Igf1r and α-SMA) in each time points. Scale bars, 100 μm.
